# Supplementary material for: Where to flee? Preferences for host communities among displaced people in Congo
Source: PLoS One. 2025 Dec 9;20(12):e0337530. doi: 10.1371/journal.pone.0337530 (PMC12688085; doi:10.1371/journal.pone.0337530)
Supplement: S1 Text — (PDF) [file pone.0337530.s001.pdf]

Supporting Information for:

"Where to flee? Preferences for host communities among displaced people in Congo"

S1 Appendix. Conflict and Displacement in the Greater Kasai.

S2 Appendix. Sampling Frame and Sample.

Table S2-1. Sampling Frame.

Figure S2-1. Identification of Population Groups of Interest.

Table S2-2. Households Selected for Survey.

Table S2-3. Household Sample.

Table S2-4. Selection Into the Conjoint Experiment.

S3 Appendix. Variable Definitions and Summary Information.

Table S3-1. Summary Statistics and Operationalization.

Table S3-2. Summary Statistics by Household Type.

S4 Appendix. Main Results in Tabular Format.

Table S4-1. Results in Tabular Format for Figure 3.

Table S4-2. Results in Tabular Format for Figure 4.

Table S4-3. Results in Tabular Format for Figure 5.

Table S4-4. Difference in Attribute Effect Sizes for Figure 3.

Table S4-5. Difference in Attribute Effect Sizes for Figure 5.

S5 Appendix. Interactions between Treatment Arms.

Figure S5-1. Preferences for Host Community with Interactions.

S6 Appendix. Robustness Tests.

Figure S6-1. Marginal Means for Host Community Choice (Figure 3).

Table S6-1. Full Numeric Results for Figure S6-1.

Figure S6-2. Marginal Means for Host Community Choice by Displacement Experience (Figure 5).

Table S6-2. Full Numeric Results for Figure S6-2.

Figure S6-3. Preferences for Host Community (Figure 3) using Continuous Outcome.

Table S6-3. Results in Tabular Format for Figure S6-3.

Figure S6-4. Results Figure 3 Controlling for Covariates.

Figure S6-5. Results Figure 5 Controlling for Covariates.

Figure S6-6. Preferences for Host Community (Figure 3) by Tshiluba speakers.

Figure S6-7. Preferences for Host Community (Figure 3) by Respondent Literacy.

S1 Appendix. Conflict and Displacement in the Greater Kasai.

The conflict in the Greater Kasai can be traced back to a disagreement over traditional authority [1].[1] Since his return in 2012 from South Africa, Jean-Pierre Pandi claimed the succession as *Kamwina Nsapu*, one of the main customary chiefs in Dibaya territory in Kasai-Central. These positions require recognition from Congo's central state. The central government in Kinshasa under President Joseph Kabila refused to officially recognize Pandi, in part because the Greater Kasai region is an opposition stronghold [1].

In August 2016, after the tone between the government and Pandi had already become increasingly belligerent, Pandi was killed in clashes between his fighters and state security forces. The incident escalated the violence, and Pandi's supporters – known as *Kamwina Nsapu* militia - intensified their attacks against the state. The conflict spread to other provinces, including Kasai and Kasai-Oriental, with the most intense fighting taking place in the first half of 2017. Both sides of the conflict have been condemned for their brutality toward civilians. The *Kamwina Nsapu* recruited heavily amongst children, burnt houses and induced large-scale displacement. At the same time, government troops used excessive violence against militia and civilians considered sympathetic to *Kamwina Nsapu* [3, 4].

The conflict is estimated to have caused the displacement of more than 1.4 million people within the Democratic Republic of the Congo and about 35,000 refugees seeking safety in Angola [5]. The vast majority of the displaced did not seek safety in camps or larger cities but took refuge with host families in rural settlements [6, 7]. After July 2017, active fighting decreased and people started returning home in some areas, while pockets of insecurity remained [4].

To this day, displaced populations and their host communities face significant challenges. The conflict has further deepened the chronic food insecurity that has characterized the region for decades. In 2025, an estimated 3.3 million people, around 26% of the Kasai population, are projected to face an acute food and livelihood crisis or worse [8]. In addition, ethnic tensions remain, leading to additional instances of violence and displacement (e.g., [9]).

[1] See, e.g., [2] for a historical overview of the Kasai conflict.

S2 Appendix. Sampling Frame and Sample.

The survey aimed to collect data from i) internally displaced people (IDPs), ii) returned IDPs, iii) repatriated refugees and iv) members of the host community in areas of Congo's Kasai, Kasai Central and Kasai Oriental provinces that are particularly affected by displacement. The survey was commissioned and overseen by UNHCR, implemented by the Congolese National Institute of Statistics (INS), and designed by UNHCR in collaboration with the research team. In Kasai, displaced people do not live in camps, but are hosted by communities (UNHCR, personal communication, 2021). Since there is no complete and up-to-date list of villages and towns in the Kasai provinces, we created a sampling frame, and sample, using the following steps.

1. **Selection of Health Zones.** The provinces Kasai, Kasai Oriental and Kasai Central consist of a total of 63 health zones. Prior to the survey, the UNHCR and INS had information on the estimated number of IDPs in 2019 and returned IDPs in 2019 per health zone, and whether the health zone included repatriated refugees. In addition, we obtained information on dwelling locations from Google AI (discussed below). Together with the UNHCR, we purposefully selected the 27 health zones in the Kasai provinces most affected by displacement. We did so as follows. We calculated the number of dwellings per health zone. Together with the UNHCR, we decided to work only in those health zones where the number of IDPs plus the number of returned IDPs in 2019 was larger than the number of dwellings. In total, 27 of the 63 health zones met this criterion.

2. **Selection of Localities.** For the three Kasai provinces, like other Congolese provinces, there is no complete and up-to-date list of villages and towns. In response, we made use of all dwellings as mapped by Google AI.[2] We overlaid these data with a 1x1km2 grid and identified 5,302 "localities": grid cells with at least 100 dwellings. We sampled localities proportional to population size in two stages: i) we determined the number of localities to select within a health zone proportional to the health zone's number of dwellings, and subsequently ii) within the health zone, localities were selected proportional to locality dwelling numbers. In total, we randomly selected 126 localities.

3. **Listing Exercise.** Between July and November 2022, we conducted a listing survey, targeting all households in all 126 localities. We undertook this exercise to facilitate household selection. First, it allowed us to obtain a complete sampling frame of all households present in a locality. In the Kasai context, doing so is complicated because a dwelling may contain multiple households; for example, a household may host a displaced household within the dwelling. Second, we aimed to randomly select households by household type. The listing exercise classified households into four types: i) member of the host community, ii) internally displaced person (IDP), iii) returned IDP, and iv) repatriated refugee. The survey included a set of questions, created in collaboration with UNHCR, which are provided in Figure S2-1 to group households across these types.

   For logistical reasons, we were not able to visit seven localities. In total, the listing survey contains information from 119 localities, 25,295 dwellings, and 33,500 households. The distribution of the households across the four types is given in Table S2-1.

Table S2-1. Sampling Frame

Province	Localities	Host	Repatriated Refugee	Returned IDP	IDP	Total
Kasai	25	2,171	1,291	2,392	3,813	9,667
Kasai Oriental	14	360	0	1,118	81	1,559
Kasai Central	80	4,452	659	14,473	2,690	22,274
Total	119	6,983	1,950	17,983	6,583	33,500

Notes: Distribution of households across type and province. Data from listing exercise.

4. **Household and Respondent Selection.** We aimed to survey 1,000 households of each household type. Multiple localities, however, had few to no households of a certain type.[3] The household selection strategy was therefore as follows. We targeted N of each household type in each locality. If there were fewer than N households of a certain type in a locality, all were selected. If there were more, N households were randomly selected.[4] N increases until, in total, more than 1,000 households are selected for that household type. We

[2] These data are freely available online: https://sites.research.google/open-buildings

[3] This was particularly the case for repatriated refugees, who cluster geographically.

[4] In each locality, we also selected up to five replacement households for the IDP, returned IDP and member of the host community types. For the repatriated refugees, we selected up to ten replacements (as there were more of them to be surveyed in localities where they were present).

Figure S2-1. Identification of Population Groups of Interest

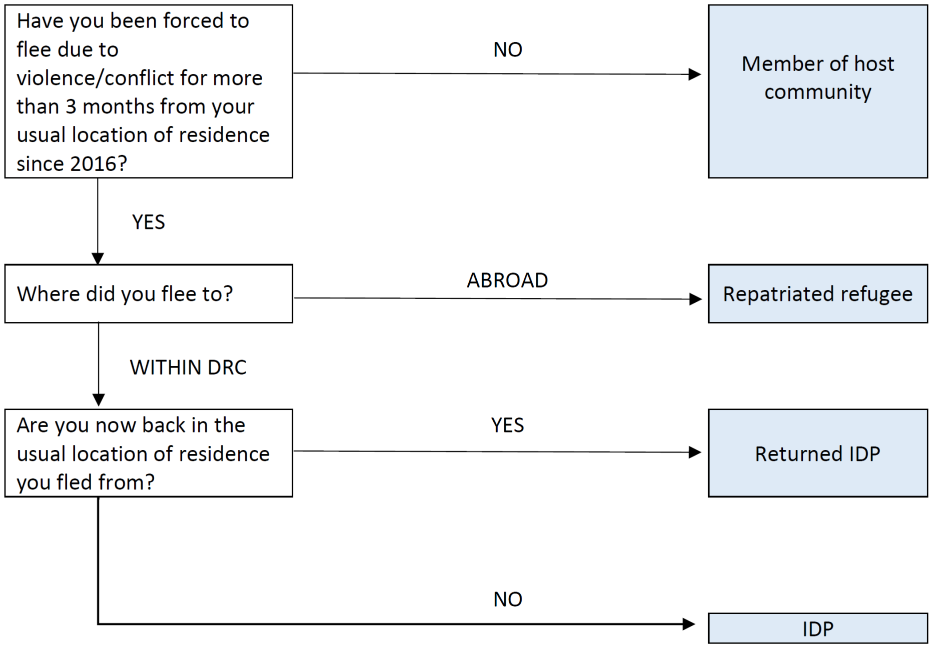

Notes: Population group definitions. Created together with UNHCR.

made use of the following numbers: N=10 for members of the host community, N=11 for IDPs, N=9 for returned IDPs, and N=29 for repatriated refugees. Table S2-2 gives an overview.

Table S2-2. Households Selected for Survey

Province	Localities	Host	Repatriated Refugee	Returned IDP	IDP	Total
Kasai	25	240	571	225	265	1,301
Kasai Oriental	14	84	0	126	64	274
Kasai Central	80	684	433	720	679	2,516
Total	119	1,008	1,004	1,071	1,008	4,091

Notes: Distribution of selected households across type and province. Data from listing exercise.

After household selection, survey teams returned to selected localities to visit the selected households. Next, the surveyor aimed to interview the head of household. If the household head was absent, the spouse of the household head was to be interviewed. When both the household head and spouse were absent, a knowledgeable adult household member would be interviewed.

5. **Sample.** Between November 2022 and February 2023, data were collected from 4,069 households across all 119 units - implying an attrition rate of 1.3%. Overall, enumerators were able to survey the sampled households; only in 3.5% of cases was it necessary to resort to surveying the pre-selected replacement households. In 64% of cases, the respondent was the household head, 27% the spouse, and 9% other. Table S2-3 gives an overview of households interviewed by province and type, where household type is based on information from the household survey.

   There are some discrepancies between household types in the listing exercise and survey (see Table S2-2 versus Table S2-3). For example, fewer repatriated refugee and returned IDP households were interviewed than targeted, but more members of the host community were interviewed. This is because the listing exercise collected only basic information at the household level, while the survey collected detailed displacement information on all household members, allowing a finer categorization. In our analysis, we focus on whether the *respondent* reports being a host community member, returned IDP, repatriated refugee or IDP.

6. **Sample and Selection into Conjoint Experiment.** Finally, not all respondents participated in the

Table S2-3. Household Sample

Province	Localities	Host	Repatriated Refugee	Returned IDP	IDP	Total
Kasai	25	457	311	232	300	1,300
Kasai Oriental	14	102	0	87	84	273
Kasai Central	80	974	238	674	610	2,496
Total	119	1,533	549	993	994	4,069

Notes: Distribution of interviewed households across type and province. Household type based on survey respondent.

conjoint experiment. By design, we aimed to conduct the experiment with half of the sample: only with the IDP and repatriated refugee households. Assignment to the experiment, however, was automated in the survey tool, and based on the household type from the listing exercise. In our analysis, we define household type based on the survey data, leading to a situation where households from all four types participated in the conjoint experiment: 26.06% of the host community, 22.09% of internally displaced, 39.44% of repatriated refugees, and 11.76% of returned IDPs. In Table S2-4 we show that repatriated refugees and internally displaced persons are more likely to get the assigned to the conjoint experiment, as this was the targeted group. Beyond this, individuals assigned to the experiment are slightly more likely to have no malaria and slightly less likely to be illiterate. However, the substantive effect sizes are small. We hence assume that there is no apparent selection into who has received the conjoint experiment, or not.

Table S2-4. Selection Into the Conjoint Experiment

	Conjoint Experiment
IDP	0.074*
	(0.018)
Repatriated refugee	0.608*
	(0.019)
Returned IDP	-0.070*
	(0.019)
Food insecurity	0.000
	(0.001)
Trust	-0.001
	(0.002)
No malaria	0.033*
	(0.015)
Not married	0.016
	(0.016)
Male	-0.011
	(0.015)
Never went to school	0.003
	(0.026)
Has regular income	0.006
	(0.020)
Below median levels of PTSD	0.024
	(0.015)
Cannot write or read	-0.048*
	(0.021)
Has worked in past week	-0.027
	(0.015)
(Intercept)	0.358*
	(0.028)
Num.Obs.	3987
R2	0.252
R2 Adj.	0.249
AIC	4656.7
BIC	4751.1
Log.Lik.	-2313.357
RMSE	0.43

Notes: Respondent selection into the conjoint experiment.

S3 Appendix. Variable Definitions and Summary Information.

Table S3-1 presents variable definitions and summary information for all variables used in this study. Table S3-2 presents summary information by household displacement status, including a test of difference across groups.

Table S3-1. Summary Statistics and Operationalization

Variable	Type	Q.	Obs.	Median	Mean	St.Dev.	Min	Max
Displacement experience								
Host community	Binary. Whether respondent is host community (1) or not (0)	s02q01	1952	0	0.262	0.440	0	1
IDP household	Binary. Whether respondent is IDP (1) or not (0)	s02q01, s02q06, s02q07	1952	0	0.222	0.416	0	1
Repatriated refugee	Binary. Whether respondent is repatriated refugee (1) or not (0)	s02q01, s02q06	1952	0	0.397	0.489	0	1
Returned IDP	Binary. Whether respondent is returned IDP (1) or not (0)	s02q01, s02q06, s02q07	1952	0	0.118	0.323	0	1
Currently hosting	Binary. Whether household is currently hosting displaced persons (1) or not (0)	s01q00b1	1965	0	0.166	0.373	0	1
Demographics								
Married	Binary. Whether respondent is married	s01q07	1965	1	0.730	0.444	0	1
Male	Binary. Whether main respondent is male	s01q02	1965	0	0.495	0.500	0	1
Attended school	Binary. Whether respondent ever attended school	s03q01	1965	1	0.877	0.328	0	1
Ever graduated	Binary. Whether respondent has at least completed primary school	s03q04	1965	1	0.736	0.441	0	1
Literacy	Binary. Whether respondent can read and write	s03q13	1965	1	0.770	0.421	0	1
Socio-economic situation								
Working	Binary. Whether respondent has been economically active in own businesses, agriculture, employment, or apprenticeships in the past 7 days	s08q05	1965	1	0.502	0.500	0	1
Regular income	Binary. Whether respondent reports having a regular income or not	s11q12	1965	0	0.148	0.355	0	1
Food insecurity	Continuous (0-35). WFP's Reduced Coping Strategies Index. Number of days in the past 7 days that if there was not enough food or money to buy food, how often the household had to: 1) rely on less preferred food, 2) borrow food, 3) reduce meals, 4) limit portion sizes, 5) restrict adult food consumption. Each item scores 0-7. Scores are summed to produce the index.	s05q01	1965	11	12.000	8.610	0	35
Food insecurity	Binary. Whether the food insecurity is above or below the median.	s05q01	1965	0	0.474	0.499	0	1
Physical and mental health								
PTSD	Binary. Whether respondent scores above the median PTSD score (1) or not (0). Used in subgroup analysis	s04q18	1965	0	0.367	0.482	0	1
Positive malaria test	Binary. Whether respondent tested positive for malaria (1) or not (0) using the WHO-validated standard diagnostics malaria antigen P.f (HRP-II) rapid test.	s04q14	1965	0	0.311	0.463	0	1
Social cohesion and experiences of violence								
Trust across groups (numeric)	Continuous (0-10). Sum of ten binary trust variables for trust in: family, neighbors, other ethnicity, other nationalities, other political affiliations, other religions, and other households in the dwelling, trust in refugees, in hosts, and in returnees	s15q03	1965	6	5.450	3.570	0	10
Experienced violence	Binary. Whether household has experienced property destruction, physical violence, theft, kidnapping, armed conflict, verbal threats, forced labor, sexual violence, conflict-induced injury, domestic violence, rape witnessing, killing of household member, forced recruitment, displacement, extortion or other forms of violence in the past 12 months	s13q14	1965	0	0.115	0.319	0	1
Concerned about violence	Binary. Whether respondent is currently concerned about criminal violence, inter-communal violence, violence against women, against refugees/displaced persons, armed conflict, terrorism, natural disaster, poverty, unemployment, hunger, expulsion, child trafficking, external aggression or robbers	s13q11	1965	1	0.849	0.358	0	1
Community meeting attendance	Binary. Whether respondent head attended community meetings in last year (1) or not (0)	s14q05ba	1965	0	0.256	0.437	0	1
Ethnic and religious identity								
Tshiluba speaker	Binary. Whether respondent's mother tongue is Tshiluba	s01q10	1965	1	0.677	0.468	0	1
Bubindi speaker	Binary. Whether respondent's mother tongue is Bubindi	s01q10	1965	0	0.114	0.318	0	1
Tshikete speaker	Binary. Whether respondent's mother tongue is Tshikete	s01q10	1965	0	0.034	0.182	0	1
Lingala speaker	Binary. Whether respondent's mother tongue is Lingala	s01q10	1965	0	0.037	0.189	0	1
Any Christian denomination	Binary. Whether the respondent belongs to any Christian domination or not	s01q12	1965	1	0.890	0.314	0	1

Variable	Type	Q.	Obs.	Median	Mean	St.Dev.	Min	Max
Protestant	Binary. Whether respondent is Protestant	s01q12	1965	0	0.228	0.420	0	1
Catholic	Binary. Whether respondent is Catholic	s01q12	1965	0	0.154	0.361	0	1
Revivalist	Binary. Whether respondent is Revivalist	s01q12	1965	0	0.250	0.433	0	1
Apostolic	Binary. Whether respondent is New Apostolic	s01q12	1965	0	0.104	0.305	0	1
Kimbanguist	Binary. Whether respondent is Kimbanguist	s01q12	1965	0	0.047	0.212	0	1
Animist	Binary. Whether respondent is Animist	s01q12	1965	0	0.035	0.183	0	1
Muslim	Binary. Whether respondent is Muslim	s01q12	1965	0	0.038	0.192	0	1
Weekly church attendance	Binary. Whether respondent reports going to church at least once per week or more often	s14q06	1965	1	0.622	0.485	0	1

Notes: Variable definitions and summary information. "Q" indicates the question number in the survey.

Table S3-2. Summary Statistics by Household Type

	Host community						IDP						Repatriated refugee						Returned IDP						F.test	Sig.
Variable	Obs.	Median	Mean	St.Dev.	Min	Max	Obs.	Median	Mean	St.Dev.	Min	Max	Obs.	Median	Mean	St.Dev.	Min	Max	Obs.	Median	Mean	St.Dev.	Min	Max		
Food insecurity (numeric)	512	11	12.800	8.900	0	35	434	12	13.100	8.640	0	35	775	10	10.900	8.500	0	35	231	11	12.100	7.800	0	33	0.000	*
Food insecurity (binary)	512	0	0.498	0.500	0	1	434	1	0.535	0.499	0	1	775	0	0.419	0.494	0	1	231	0	0.494	0.501	0	1	0.001	*
PTSD	512	0	0.396	0.490	0	1	434	0	0.318	0.466	0	1	775	0	0.391	0.488	0	1	231	0	0.312	0.464	0	1	0.010	*
Trust across groups (numeric)	512	6	5.500	3.690	0	10	434	5	5.180	3.690	0	10	775	6	5.360	3.420	0	10	231	7	6.220	3.490	0	10	0.003	*
Experienced violence	512	0	0.107	0.310	0	1	434	0	0.115	0.320	0	1	775	0	0.116	0.321	0	1	231	0	0.126	0.332	0	1	0.908	
Concerned about violence	512	1	0.828	0.378	0	1	434	1	0.866	0.341	0	1	775	1	0.862	0.345	0	1	231	1	0.827	0.379	0	1	0.198	
Positive malaria test	512	0	0.295	0.456	0	1	434	0	0.309	0.463	0	1	775	0	0.308	0.462	0	1	231	0	0.368	0.483	0	1	0.248	
Married	512	1	0.756	0.430	0	1	434	1	0.680	0.467	0	1	775	1	0.726	0.446	0	1	231	1	0.779	0.416	0	1	0.017	*
Male	512	0	0.451	0.498	0	1	434	0	0.479	0.500	0	1	775	1	0.520	0.500	0	1	231	1	0.545	0.499	0	1	0.034	*
Regular income	512	0	0.158	0.365	0	1	434	0	0.113	0.317	0	1	775	0	0.169	0.375	0	1	231	0	0.121	0.327	0	1	0.034	*
Currently hosting	512	0	0.170	0.376	0	1	434	0	0.175	0.381	0	1	775	0	0.177	0.382	0	1	231	0	0.108	0.311	0	1	0.089	
Community meeting attendance	512	0	0.266	0.442	0	1	434	0	0.200	0.401	0	1	775	0	0.257	0.437	0	1	231	0	0.338	0.474	0	1	0.002	*
Attended school	512	1	0.848	0.360	0	1	434	1	0.834	0.372	0	1	775	1	0.924	0.265	0	1	231	1	0.870	0.337	0	1	0.000	*
Ever graduated	512	1	0.711	0.454	0	1	434	1	0.680	0.467	0	1	775	1	0.777	0.417	0	1	231	1	0.753	0.432	0	1	0.001	*
Literacy	512	1	0.734	0.442	0	1	434	1	0.735	0.442	0	1	775	1	0.817	0.387	0	1	231	1	0.753	0.432	0	1	0.001	*
Working	512	1	0.529	0.500	0	1	434	0	0.482	0.500	0	1	775	0	0.452	0.498	0	1	231	1	0.654	0.477	0	1	0.000	*
Tshiluba speaker	512	1	0.693	0.462	0	1	434	1	0.682	0.466	0	1	775	1	0.645	0.479	0	1	231	1	0.740	0.439	0	1	0.037	*
Bubindi speaker	512	0	0.139	0.346	0	1	434	0	0.147	0.355	0	1	775	0	0.0865	0.281	0	1	231	0	0.095	0.294	0	1	0.002	*
Tshikete speaker	512	0	0.059	0.235	0	1	434	0	0.025	0.157	0	1	775	0	0.013	0.113	0	1	231	0	0.061	0.239	0	1	0.000	*
Lingala speaker	512	0	0.022	0.145	0	1	434	0	0.044	0.205	0	1	775	0	0.056	0.229	0	1	231	0	0.000	0.000	0	0	0.000	*
Any Christian denomination	512	1	0.906	0.292	0	1	434	1	0.892	0.311	0	1	775	1	0.877	0.328	0	1	231	1	0.900	0.300	0	1	0.406	
Protestant	512	0	0.188	0.391	0	1	434	0	0.221	0.416	0	1	775	0	0.270	0.444	0	1	231	0	0.203	0.403	0	1	0.004	*
Catholic	512	0	0.164	0.371	0	1	434	0	0.173	0.379	0	1	775	0	0.141	0.348	0	1	231	0	0.147	0.355	0	1	0.440	
Revivalist	512	0	0.256	0.437	0	1	434	0	0.221	0.416	0	1	775	0	0.255	0.436	0	1	231	0	0.268	0.444	0	1	0.466	
Apostolic	512	0	0.115	0.320	0	1	434	0	0.085	0.280	0	1	775	0	0.093	0.290	0	1	231	0	0.147	0.355	0	1	0.046	*
Kimbanguist	512	0	0.049	0.216	0	1	434	0	0.062	0.242	0	1	775	0	0.045	0.208	0	1	231	0	0.022	0.146	0	1	0.131	
Animist	512	0	0.041	0.199	0	1	434	0	0.035	0.183	0	1	775	0	0.027	0.162	0	1	231	0	0.039	0.194	0	1	0.557	
Muslim	512	0	0.025	0.157	0	1	434	0	0.039	0.194	0	1	775	0	0.048	0.213	0	1	231	0	0.030	0.172	0	1	0.200	
Weekly church attendance	512	1	0.596	0.491	0	1	434	1	0.664	0.473	0	1	775	1	0.652	0.477	0	1	231	1	0.515	0.501	0	1	0.000	*

Notes: Comparison of population groups in the study sample. Statistical significance markers: * $p<0.05$

S4 Appendix. Main Results in Tabular Format.

Table S4-1, Table S4-2, and Table S4-3 gives the results from Figures 3, 4 and 5 in tabular format. Table S4-4 presents a formal test of differences in effect sizes across attributes for Figure 3, and Table S4-5 does so for Figure 5.

Table S4-1. Results in Tabular Format for Figure 3

Conjoint level	AMCE	Std. Error	Z	Lower (α=0.05)	Upper (α=0.05)	Lower (α=0.1)	Upper (α=0.1)
Able to get work in community	0.105	0.012	9.100	0.082	0.127	0.086	0.124
Able to join local church	0.068	0.011	5.951	0.045	0.090	0.049	0.086
Has relatives in the community	0.059	0.012	5.129	0.036	0.082	0.040	0.078
Community speaks mother tongue	0.041	0.011	3.610	0.019	0.063	0.022	0.060
Attend village meetings	0.052	0.012	4.475	0.029	0.074	0.033	0.071

Notes: N = 7,860. Results in tabular format for Figure 3.

Table S4-2. Results in Tabular Format for Figure 4

Outcome	Conjoint level	AMCE	Std. error	Z	P	Lower (α=0.05)	Lower (α=0.1)	Upper (α=0.05)	Upper (α=0.1)
Economic contribution	Able to get work in community	0.766	0.053	14.587	0.000	0.663	0.680	0.869	0.852
Economic contribution	Able to join local church	0.192	0.047	4.070	0.000	0.099	0.114	0.284	0.269
Economic contribution	Has relatives in the community	0.154	0.048	3.186	0.001	0.059	0.075	0.249	0.234
Economic contribution	Community speaks mother tongue	0.071	0.049	1.453	0.146	-0.025	-0.009	0.166	0.151
Economic contribution	Attend village meetings	0.185	0.050	3.740	0.000	0.088	0.104	0.282	0.267
Feeling welcome	Able to get work in community	0.389	0.048	8.100	0.000	0.295	0.310	0.483	0.468
Feeling welcome	Able to join local church	0.352	0.046	7.677	0.000	0.262	0.277	0.442	0.427
Feeling welcome	Has relatives in the community	0.233	0.046	5.006	0.000	0.142	0.156	0.324	0.309
Feeling welcome	Community speaks mother tongue	0.150	0.046	3.238	0.001	0.059	0.074	0.241	0.227
Feeling welcome	Attend village meetings	0.372	0.048	7.734	0.000	0.278	0.293	0.466	0.451
Feeling safe	Able to get work in community	0.383	0.047	8.204	0.000	0.292	0.307	0.475	0.460
Feeling safe	Able to join local church	0.318	0.045	7.131	0.000	0.230	0.244	0.405	0.391
Feeling safe	Has relatives in the community	0.286	0.047	6.058	0.000	0.194	0.208	0.379	0.364
Feeling safe	Community speaks mother tongue	0.187	0.047	3.993	0.000	0.095	0.110	0.279	0.264
Feeling safe	Attend village meetings	0.368	0.047	7.786	0.000	0.276	0.290	0.461	0.446
Contribute ideas	Able to get work in community	0.250	0.049	5.138	0.000	0.155	0.170	0.346	0.331
Contribute ideas	Able to join local church	0.212	0.047	4.498	0.000	0.120	0.135	0.304	0.290
Contribute ideas	Has relatives in the community	0.115	0.048	2.384	0.017	0.020	0.036	0.210	0.194
Contribute ideas	Community speaks mother tongue	0.145	0.048	3.004	0.003	0.050	0.066	0.240	0.225
Contribute ideas	Attend village meetings	0.691	0.053	13.156	0.000	0.588	0.605	0.794	0.778
Trust community	Able to get work in community	0.411	0.049	8.479	0.000	0.316	0.332	0.507	0.491
Trust community	Able to join local church	0.220	0.045	4.917	0.000	0.132	0.147	0.308	0.294
Trust community	Has relatives in the community	0.157	0.048	3.290	0.001	0.063	0.078	0.250	0.235
Trust community	Community speaks mother tongue	0.088	0.047	1.870	0.062	-0.004	0.011	0.181	0.166
Trust community	Attend village meetings	0.357	0.049	7.248	0.000	0.260	0.276	0.453	0.437

Notes: N = 7,860. Results in tabular format for Figure 4.

Table S4-3. Results in Tabular Format for Figure 5

Population group	Conjoint level	AMCE	Std. error	Z	P	Lower (α=0.05)	Lower (α=0.1)	Upper (α=0.05)	Upper (α=0.1)
Host community	Able to get work in community	0.071	0.023	3.150	0.002	0.027	0.034	0.115	0.108
Host community	Able to join local church	0.050	0.021	2.360	0.018	0.008	0.015	0.091	0.085
Host community	Has relatives in the community	0.027	0.022	1.199	0.231	-0.017	-0.010	0.070	0.063
Host community	Community speaks mother tongue	0.061	0.022	2.724	0.006	0.017	0.024	0.105	0.098
Host community	Attend village meetings	0.037	0.023	1.642	0.101	-0.007	0.000	0.081	0.074
IDP	Able to get work in community	0.154	0.024	6.328	0.000	0.106	0.114	0.202	0.194
IDP	Able to join local church	0.118	0.025	4.747	0.000	0.069	0.077	0.166	0.159
IDP	Has relatives in the community	0.045	0.024	1.850	0.064	-0.003	0.005	0.092	0.084
IDP	Community speaks mother tongue	0.038	0.025	1.568	0.117	-0.010	-0.002	0.086	0.079
IDP	Attend village meetings	0.046	0.025	1.866	0.062	-0.002	0.005	0.094	0.086
Repatriated refugee	Able to get work in community	0.124	0.018	6.724	0.000	0.088	0.093	0.160	0.154
Repatriated refugee	Able to join local church	0.059	0.018	3.281	0.001	0.024	0.030	0.095	0.089
Repatriated refugee	Has relatives in the community	0.096	0.019	5.101	0.000	0.059	0.065	0.133	0.127
Repatriated refugee	Community speaks mother tongue	0.048	0.018	2.699	0.007	0.013	0.019	0.084	0.078
Repatriated refugee	Attend village meetings	0.055	0.019	2.966	0.003	0.019	0.024	0.091	0.085
Returned IDP	Able to get work in community	0.032	0.032	0.990	0.322	-0.031	-0.021	0.095	0.085
Returned IDP	Able to join local church	0.044	0.034	1.274	0.203	-0.024	-0.013	0.111	0.100
Returned IDP	Has relatives in the community	0.032	0.033	0.981	0.327	-0.032	-0.022	0.096	0.086
Returned IDP	Community speaks mother tongue	-0.011	0.032	-0.337	0.736	-0.074	-0.064	0.053	0.042
Returned IDP	Attend village meetings	0.078	0.032	2.405	0.016	0.014	0.025	0.142	0.131

Notes: N = 7,860. Results in tabular format for Figure 5.

Table S4-4. Difference in Attribute Effect Sizes for Figure 3

	Job availability (Economic)	Church membership (Social)	Family ties (Relational)	Language ties (Cultural)	Village decisions (Political)
Job availability (Economic integration)	.				
Church membership (Social integration)	0.022*	.			
Family ties (Relational integration)	0.005*	0.599	.		
Language ties (Cultural integration)	0.000*	0.100	0.268	.	
Village decisions (Political integration)	0.001*	0.326	0.65	0.517	.

Notes: Formal test of difference between AMCEs based on pairwise t-tests between all attributes for Figure 3.

Table S4-5. Difference in Attribute Effect Sizes for Figure 5

Population group	Level	Diff in AMCE	Std.error	Z	P	Lower	Upper
IDP - Host community	Able to get work in community	0.083	0.033	2.509	0.012	0.018	0.148
Repatriated refugee - Host community	Has relatives in the community	0.070	0.029	2.398	0.017	0.013	0.127
IDP - Host community	Able to join local church	0.068	0.033	2.083	0.037	0.004	0.132

Notes: Formal test of difference between AMCEs based on pairwise t-tests between all attributes for Figure 5.
Only significant differences are displayed.

S5 Appendix. Interactions between Treatment Arms.
Figure S5-1 presents the five main effects and the ten (=5*(5-1)/2) interactions between treatment arms.

Figure S5-1. Preferences for Host Community with Interactions

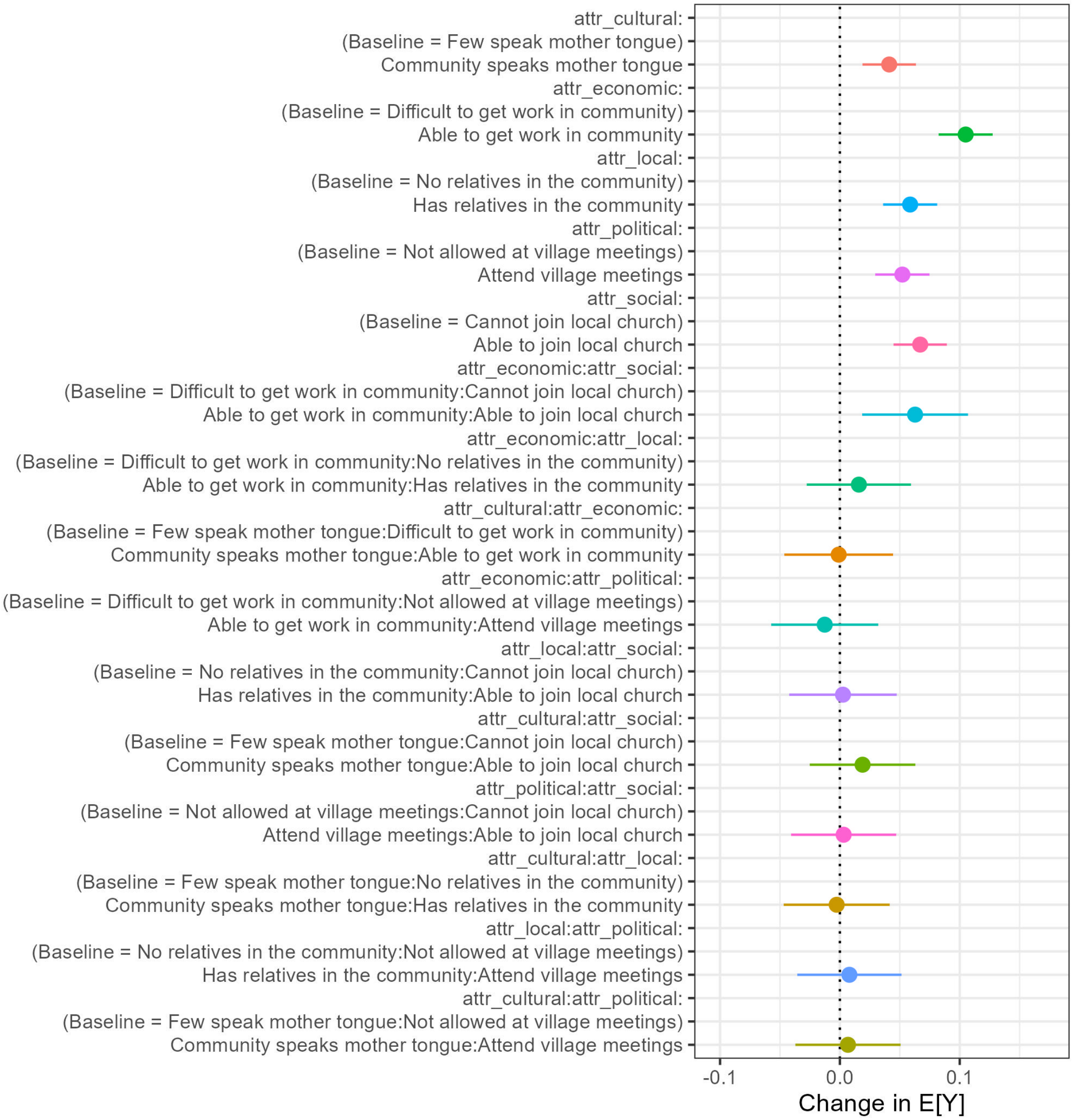

Notes: Average Marginal Component Effects for Host Community Choice (N = 7,860). Dots are the AMCE point estimates and horizontal lines show 95% confidence intervals.

S6 Appendix. Robustness Tests.

Results using Marginal Means.

Figure S6-1 provides the marginal means for Figure 3, with results in tabular format in Table S6-1. Figure S6-2 provides the marginal means for Figure 5, with results in tabular format in Table S6-2.

Figure S6-1. Marginal Means for Host Community Choice (Figure 3)

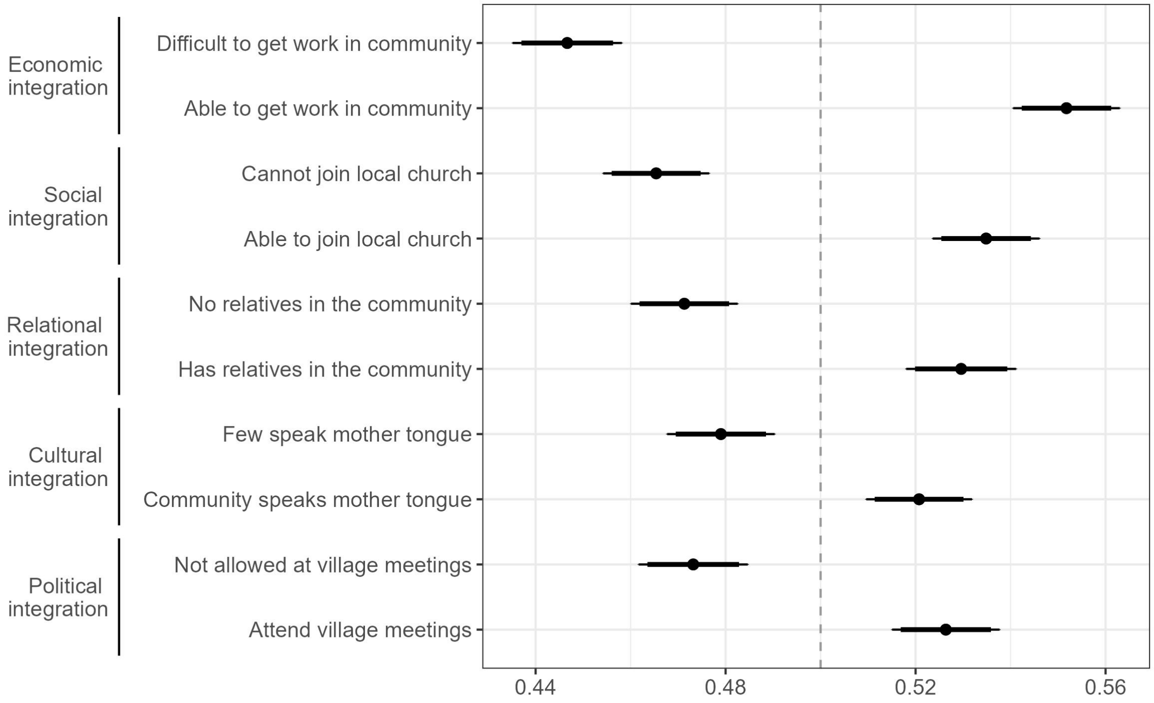

Notes: Marginal means effects for host community choice (N= 7,860). Dots are the marginal means point estimates and thin (thick) horizontal lines show 95% (90%) confidence intervals.

Table S6-1. Full Numeric Results for Figure S6-1

Conjoint level	Marginal mean	Std. Error	Z	Lower (α=0.05)	Upper (α=0.05)	Lower (α=0.1)	Upper (α=0.1)
Difficult to get work in community	0.447	0.006	76.171	0.435	0.437	0.458	0.456
Able to get work in community	0.552	0.006	96.393	0.541	0.542	0.563	0.561
Cannot join local church	0.465	0.006	81.743	0.454	0.456	0.477	0.475
Able to join local church	0.535	0.006	93.250	0.524	0.525	0.546	0.544
No relatives in the community	0.471	0.006	82.247	0.460	0.462	0.483	0.481
Has relatives in the community	0.530	0.006	89.765	0.518	0.520	0.541	0.539
Few speak mother tongue	0.479	0.006	82.949	0.468	0.470	0.490	0.489
Community speaks mother tongue	0.521	0.006	91.662	0.510	0.511	0.532	0.530
Not allowed at village meetings	0.473	0.006	80.738	0.462	0.464	0.485	0.483
Attend village meetings	0.526	0.006	91.215	0.515	0.517	0.538	0.536

Notes: N = 7,860. Results in tabular format for Figure S6-1.

Figure S6-2. Marginal Means for Host Community Choice (Figure 5), by Displacement Experience

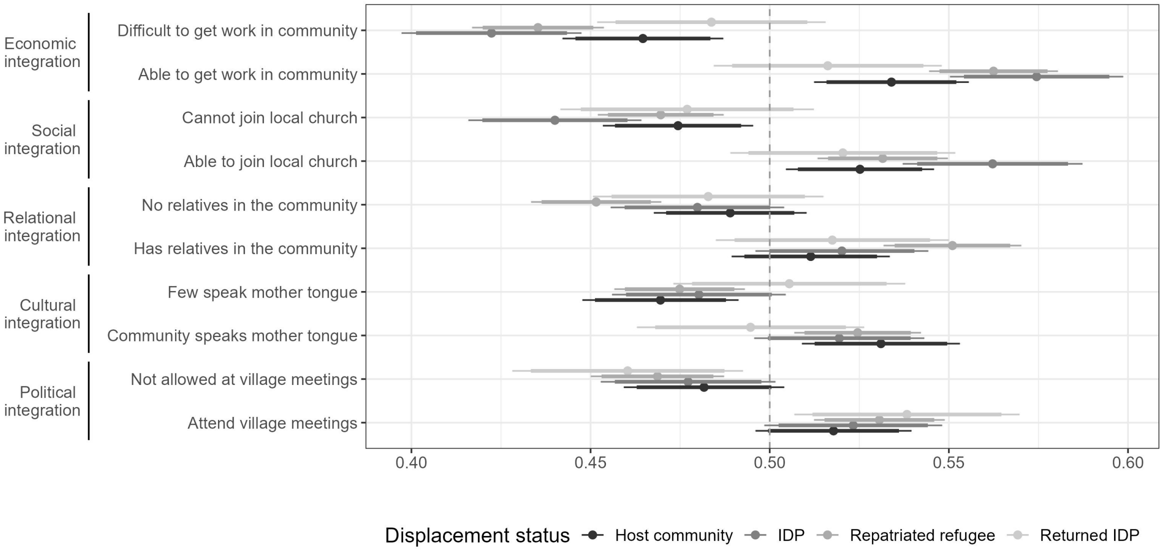

Notes: Marginal means effects for host community choice (N= 7,860). Dots are the marginal means point estimates and thin (thick) horizontal lines show 95% (90%) confidence intervals.

Table S6-2. Full Numeric Results for Figure S6-2

Population group	Conjoint level	Marginal	Std.	Z	Lower	Upper	Lower	Upper
	mean	Error		(α=0.05)	(α=0.05)	(α=0.1)	(α=0.1)	
Host community	Difficult to get work in community	0.465	0.011	40.592	0.442	0.446	0.487	0.483
Host community	Able to get work in community	0.534	0.011	48.488	0.512	0.516	0.556	0.552
Host community	Cannot join local church	0.474	0.011	44.344	0.453	0.457	0.495	0.492
Host community	Able to join local church	0.525	0.011	49.815	0.505	0.508	0.546	0.543
Host community	No relatives in the community	0.489	0.011	44.967	0.468	0.471	0.510	0.507
Host community	Has relatives in the community	0.511	0.011	45.437	0.489	0.493	0.534	0.530
Host community	Few speak mother tongue	0.470	0.011	42.241	0.448	0.451	0.491	0.488
Host community	Community speaks mother tongue	0.531	0.011	47.231	0.509	0.513	0.553	0.550
Host community	Not allowed at village meetings	0.482	0.011	42.165	0.459	0.463	0.504	0.500
Host community	Attend village meetings	0.518	0.011	46.627	0.496	0.500	0.540	0.536
IDP	Difficult to get work in community	0.422	0.013	32.979	0.397	0.401	0.447	0.443
IDP	Able to get work in community	0.574	0.012	46.580	0.550	0.554	0.599	0.595
IDP	Cannot join local church	0.440	0.012	35.730	0.416	0.420	0.464	0.460
IDP	Able to join local church	0.562	0.013	43.930	0.537	0.541	0.587	0.583
IDP	No relatives in the community	0.480	0.012	38.861	0.456	0.460	0.504	0.500
IDP	Has relatives in the community	0.520	0.012	42.261	0.496	0.500	0.544	0.540
IDP	Few speak mother tongue	0.480	0.012	38.846	0.456	0.460	0.504	0.501
IDP	Community speaks mother tongue	0.519	0.012	42.898	0.496	0.499	0.543	0.539
IDP	Not allowed at village meetings	0.477	0.012	38.379	0.453	0.457	0.502	0.498
IDP	Attend village meetings	0.523	0.013	41.341	0.499	0.502	0.548	0.544
Repatriated refugee	Difficult to get work in community	0.435	0.009	46.400	0.417	0.420	0.454	0.451
Repatriated refugee	Able to get work in community	0.562	0.009	61.351	0.544	0.547	0.580	0.578
Repatriated refugee	Cannot join local church	0.470	0.009	52.427	0.452	0.455	0.487	0.484
Repatriated refugee	Able to join local church	0.532	0.009	57.315	0.513	0.516	0.550	0.547
Repatriated refugee	No relatives in the community	0.452	0.009	48.730	0.433	0.436	0.470	0.467
Repatriated refugee	Has relatives in the community	0.551	0.010	56.210	0.532	0.535	0.570	0.567
Repatriated refugee	Few speak mother tongue	0.475	0.009	51.132	0.457	0.460	0.493	0.490
Repatriated refugee	Community speaks mother tongue	0.525	0.009	58.197	0.507	0.510	0.542	0.539
Repatriated refugee	Not allowed at village meetings	0.469	0.009	49.409	0.450	0.453	0.487	0.484
Repatriated refugee	Attend village meetings	0.531	0.009	57.022	0.512	0.515	0.549	0.546
Returned IDP	Difficult to get work in community	0.484	0.016	29.746	0.452	0.457	0.516	0.510
Returned IDP	Able to get work in community	0.516	0.016	31.815	0.484	0.490	0.548	0.543
Returned IDP	Cannot join local church	0.477	0.018	26.438	0.442	0.447	0.512	0.507
Returned IDP	Able to join local church	0.520	0.016	32.501	0.489	0.494	0.552	0.547
Returned IDP	No relatives in the community	0.483	0.016	29.435	0.451	0.456	0.515	0.510
Returned IDP	Has relatives in the community	0.517	0.017	31.212	0.485	0.490	0.550	0.545
Returned IDP	Few speak mother tongue	0.505	0.017	30.631	0.473	0.478	0.538	0.533
Returned IDP	Community speaks mother tongue	0.495	0.016	30.576	0.463	0.468	0.526	0.521
Returned IDP	Not allowed at village meetings	0.460	0.016	28.030	0.428	0.433	0.493	0.487
Returned IDP	Attend village meetings	0.538	0.016	33.579	0.507	0.512	0.570	0.565

Notes: N = 7,860. Results in tabular format for Figure S6-2.

Conjoint Results for Continuous Measure.

As part of the survey, we do not only ask respondents to make a forced choice between host communities. After the choice, we also ask respondents to indicate to what extent they want to live in each host community on a Likert scale (0 = Not at all, 1 = Not really, 2 = Neither agree nor disagree, 3 = Somewhat, 4 = Very much). Figure S6-3 provides the AMCE for the conjoint experiment with a ranking of host communities rather than a forced choice (equivalent to the forced choice in Figure 3). The results in tabular format can be found in Table S6-3.

Figure S6-3. Preferences for Host Community (Figure 3) using Continuous Outcome

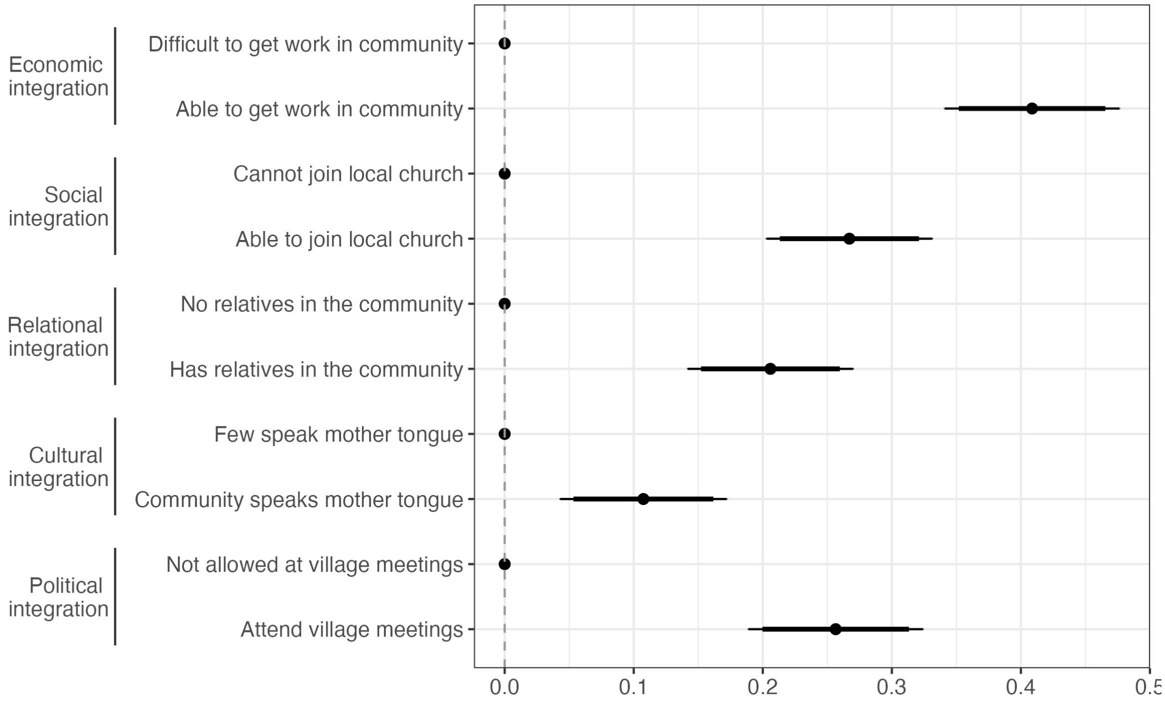

Notes: Average marginal component effects for host community preference (continuous outcome). N= 7,860. Dots are the AMCE point estimates and thin (thick) horizontal lines show 95% (90%) confidence intervals.

Table S6-3. Results in Tabular Format for Figure S6-3

Conjoint level	AMCE	Std. Error	Z	P	Lower (α=0.05)	Upper (α=0.05)	Lower (α=0.1)	Upper (α=0.1)
Able to get work in community	0.409	0.035	11.841	0.000	0.341	0.352	0.476	0.465
Able to join local church	0.267	0.033	8.150	0.000	0.203	0.213	0.331	0.321
Has relatives in the community	0.206	0.033	6.297	0.000	0.142	0.152	0.270	0.260
Community speaks mother tongue	0.108	0.033	3.264	0.001	0.043	0.053	0.172	0.162
Attend village meetings	0.257	0.035	7.434	0.000	0.189	0.200	0.324	0.313

Notes: N = 7,860. Results in tabular format for Figure S6-3.

Results Controlling for Covariates.

Figure S6-4 and Figure S6-5 replicate Figures 3 and 5 while controlling for a wide set of covariates, including respondent religion, ability to speak Tshiluba (the main local language), food insecurity status at the time of the survey, malaria diagnosis, concern about violence, marital status, gender, school attendance, literacy, educational attainment, regular income, and employment in the week prior to the survey.

Figure S6-4. Results Figure 3 Controlling for Covariates

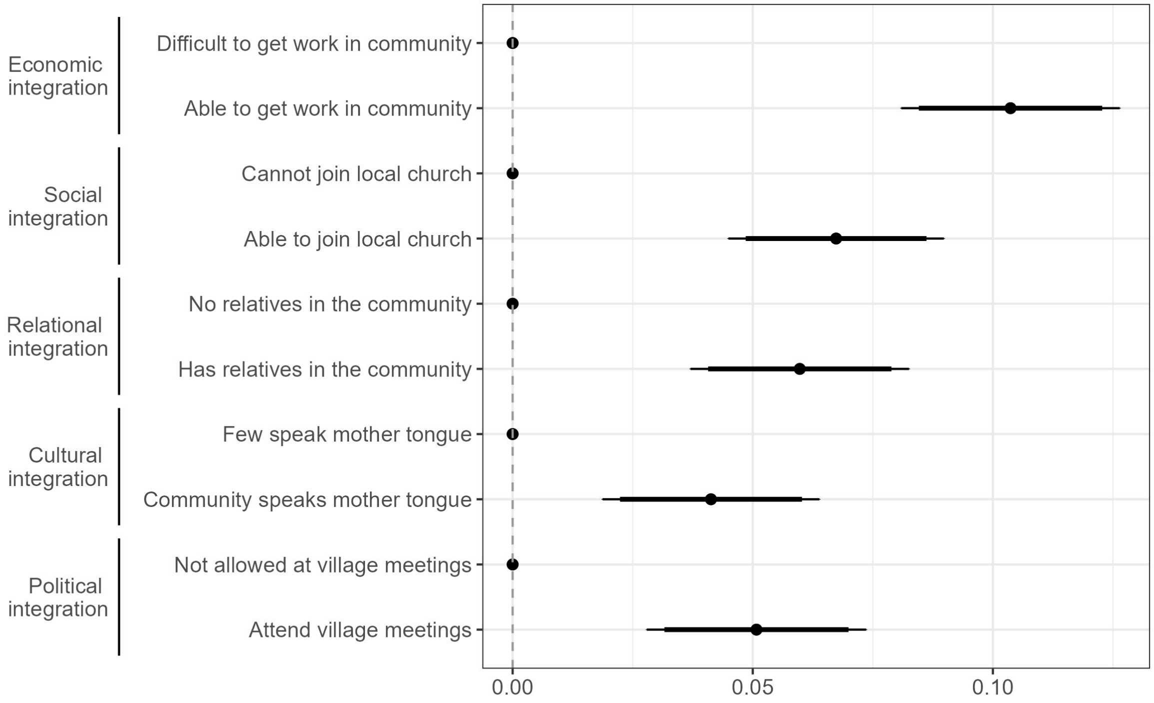

Notes: Average marginal component effects for host community preference (covariates added). Dots are the AMCE point estimates and thin (thick) horizontal lines show 95% (90%) confidence intervals.

Figure S6-5. Results Figure 5 Controlling for Covariates

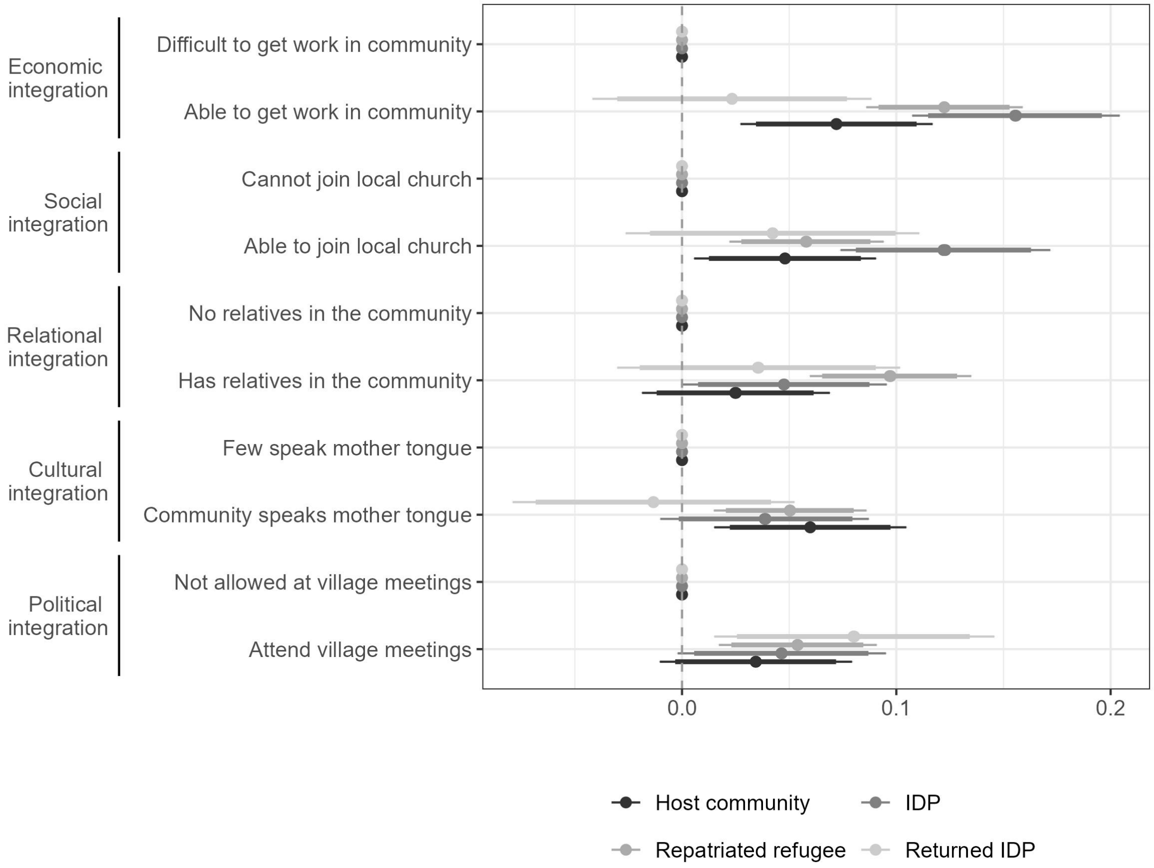

Notes: Average marginal component effects for host community preferences (covariates added) by population group. Dots are the ACME point estimates and thin (thick) horizontal lines show 95% (90%) confidence intervals.

Results by Respondent Mother Tongue.

Figure S6-6 provides results when splitting the sample into Tshiluba speakers (dominant language) and other mother tongues.

Figure S6-6. Preferences for Host Community (Figure 3) by Tshiluba speakers

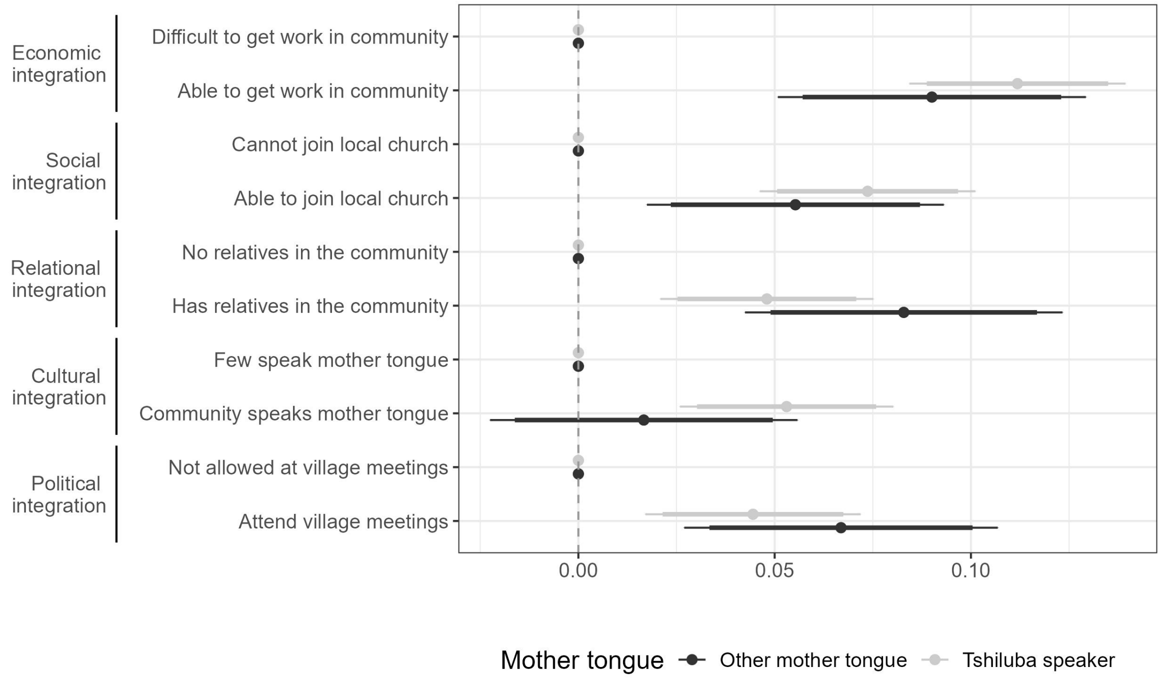

Notes: Average marginal component effects by mother tongue of respondent (N = 1,331 Tshiluba speaker, 634 non-Tshiluba speaker). Dots are the AMCE point estimates and thin (thick) horizontal lines show 95% (90%) confidence intervals.

Result by Respondent Literacy Status.

The drawings used in the experiment may highlight factors beyond the attribute level that we aim to highlight. We carefully piloted the drawings, including debriefings to ensure that respondents' interpretation of each drawing was similar to ours. Furthermore, as part of the experiment, the enumerator would place the (randomly) selected attribute levels in front of the respondent and subsequently present the profile by introducing each attribute level (while presenting the drawing) out loud.

We test here whether illiterate respondents – who maybe relied more on the drawings – differ from those who are literate. Figure S6-7, which replicates Figure 4 but separates literate and illiterate respondents, shows that there are no differences.

Figure S6-7. Preferences for Host Community (Figure 3) by Respondent Literacy

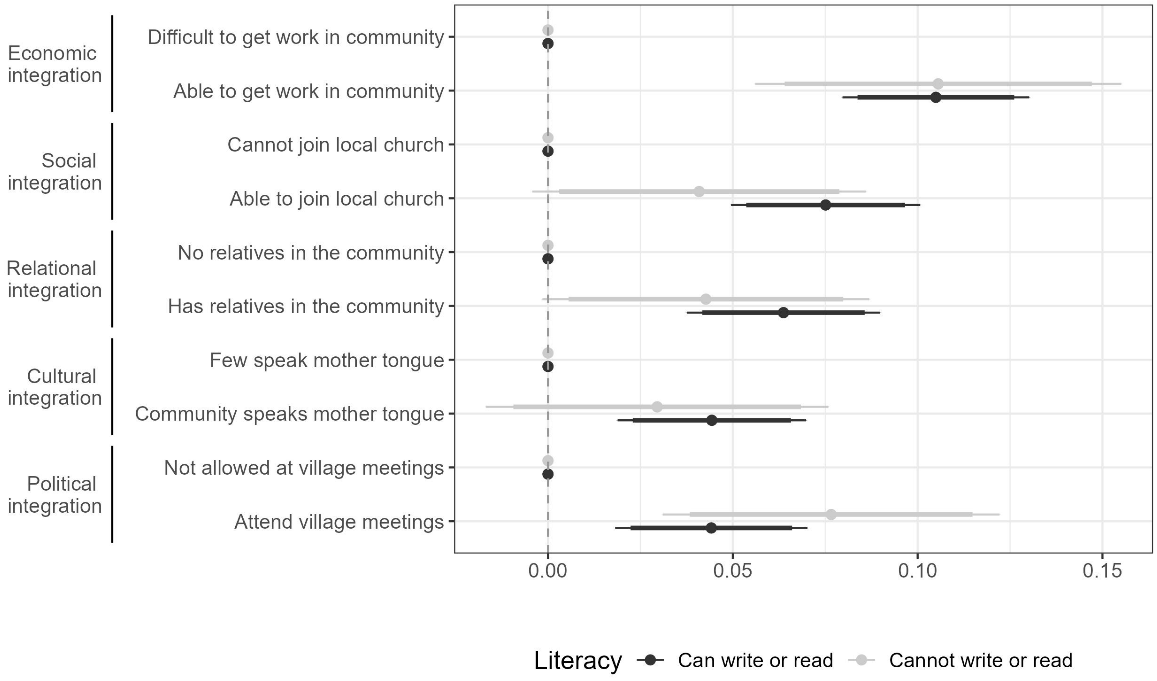

Notes: Average marginal component effects by literacy (N = 1,513 literate, 452 illiterate). Dots are the AMCE point estimates and thin (thick) horizontal lines show 95% (90%) confidence intervals.

SI References

Supporting Information References

1. Clowes W. Briefing: The conflict in Kasai, DRC; 2017. Available from: https://www.thenewhumanitarian.org/analysis/2017/07/31/briefing-conflict-kasai-drc.
2. Kabamba K. Power, Territoriality and Conflicts in Large Kasaï (Democratic Republic of Congo). Revue Belge de Géographie. 2018:1-33.
3. BBC News. DR Congo's Kasai crisis: war crimes committed by both sides, UN says.. BBC News; 2018. Available from: https://www.bbc.co.uk/news/world-africa-44613147.
4. Mercy Corps. Kasai Conflict Assessment. Mercy Corps; 2019.
5. UNHCR. Update on UNHCR's Operations in Africa; 2017.
6. Oxfam. Kasai: The forgotten Province of DRC. Oxfam; 2017.
7. Radio Okapi. Le conflit Kamuina Nsapu affecte plus d'un million de personnes (OCHA). Radio Okapi; 2017.
8. IPC. Republique Democratique du Congo: Analyse de l'insécurité alimentaire aiguë; 2025.
9. UNHCR. Violent Attacks Displace Thousands in DR Congo's Kasai Region; 2021.
