# Supplementary material for: Where to flee? Preferences for host communities among displaced people in Congo
Source: PLoS One. 2025 Dec 9;20(12):e0337530. doi: 10.1371/journal.pone.0337530 (PMC12688085; doi:10.1371/journal.pone.0337530)
Supplement: Attachment — Submitted filename: PONE-D-25-26296 cc NT20250715.pdf [file pone.0337530.s002.pdf]

PLOS ONE

Where to flee? Preferences for host communities among displaced people in Congo

--Manuscript Draft--

**Manuscript Number:**	PONE-D-25-26296
**Article Type:**	Research Article
**Full Title:**	Where to flee? Preferences for host communities among displaced people in Congo
**Short Title:**	Where to flee? Preferences for host communities among displaced people in Congo
**Corresponding Author:**	Peter van der Windt<br>New York University Abu Dhabi<br>ABU DHABI, UNITED ARAB EMIRATES
**Keywords:**	Displacement; Civil War; Civic Participation; Democratic Republic of Congo
**Abstract:**	Previous research has explored host communities' attitudes toward displaced individuals, but we know much less about what displaced people seek in a host community. Understanding both perspectives is key to fostering successful local integration. This study responds to this gap through a conjoint experiment that randomly varies the characteristics of the host community. The experiment is embedded in a survey of nearly 2,000 respondents in the Kasai region of the Democratic Republic of Congo, an area heavily affected by conflict-induced displacement. Our findings indicate that in addition to safety, displaced individuals prioritize job opportunities but also desire access to a social and political life. Our results suggest that the promotion of economic opportunities and inclusive governance are among the most effective strategies to create conditions conducive to local integration.
**Order of Authors:**	Peter van der Windt
	Nik Stoop
	Sigrid Weber
**Additional Information:**	
**Question**	**Response**
**Financial Disclosure**<br><br>Enter a financial disclosure statement that describes the sources of funding for the work included in this submission. Review the submission guidelines for detailed requirements. View published research articles from *PLOS ONE* for specific examples.<br><br>This statement is required for submission and **will appear in the published article** if the submission is accepted. Please make sure it is accurate.	Yes

Powered by Editorial Manager® and ProduXion Manager® from Aries Systems Corporation

**Funded studies**<br>Enter a statement with the following details:<br>• Initials of the authors who received each award<br>• Grant numbers awarded to each author<br>• The full name of each funder<br>• URL of each funder website<br>• Did the sponsors or funders play any role in the study design, data collection and analysis, decision to publish, or preparation of the manuscript?<br><br>Did you receive funding for this work?	
Please add funding details.<br>as follow-up to "**Financial Disclosure**<br><br>Enter a financial disclosure statement that describes the sources of funding for the work included in this submission. Review the submission guidelines for detailed requirements. View published research articles from *PLOS ONE* for specific examples.<br><br>This statement is required for submission and **will appear in the published article** if the submission is accepted. Please make sure it is accurate.<br><br>**Funded studies**<br>Enter a statement with the following details:<br>• Initials of the authors who received each award<br>• Grant numbers awarded to each author<br>• The full name of each funder<br>• URL of each funder website<br>• Did the sponsors or funders play any role in the study design, data collection and analysis, decision to publish, or preparation of the manuscript?<br><br>Did you receive funding for this work?"	NS and PW received funding from the World Bank UNHCR Joint Data Center. The funders did not play a role in the study design, data collection and analysis, decision to publish, or preparation of the manuscript
Please select the country of your main research funder (please select carefully as in some cases this is used in fee calculation).	UNITED STATES - US

Powered by Editorial Manager® and ProduXion Manager® from Aries Systems Corporation

as follow-up to "**Financial Disclosure**<br><br>Enter a financial disclosure statement that describes the sources of funding for the work included in this submission. Review the submission guidelines for detailed requirements. View published research articles from *PLOS ONE* for specific examples.<br><br>This statement is required for submission and **will appear in the published article** if the submission is accepted. Please make sure it is accurate.<br><br>**Funded studies**<br>Enter a statement with the following details:<br>• Initials of the authors who received each award<br>• Grant numbers awarded to each author<br>• The full name of each funder<br>• URL of each funder website<br>• Did the sponsors or funders play any role in the study design, data collection and analysis, decision to publish, or preparation of the manuscript?<br><br>Did you receive funding for this work?"	
**Competing Interests**<br><br>Use the instructions below to enter a competing interest statement for this submission. On behalf of all authors, disclose any competing interests that could be perceived to bias this work—acknowledging all financial support and any other relevant financial or non-financial competing interests.<br><br>This statement is required for submission and **will appear in the published article** if the submission is accepted. Please make sure it is accurate and that any funding sources listed in your Funding Information later in the submission form are also declared in your Financial Disclosure statement.	The authors have declared that no competing interests exist

View published research articles from *PLOS ONE* for specific examples.<br><br>**NO authors have competing interests**<br><br>Enter: *The authors have declared that no competing interests exist.*<br><br>**Authors with competing interests**<br><br>Enter competing interest details beginning with this statement:<br><br>*I have read the journal's policy and the authors of this manuscript have the following competing interests: [insert competing interests here]*<br><br>* typeset	
**Ethics Statement**<br><br>Enter an ethics statement for this submission. This statement is required if the study involved:<br><br>• Human participants<br>• Human specimens or tissue<br>• Vertebrate animals or cephalopods<br>• Vertebrate embryos or tissues<br>• Field research<br><br>Write "N/A" if the submission does not require an ethics statement.<br><br>General guidance is provided below. Consult the submission guidelines for detailed instructions. **Make sure that all information entered here is included in the Methods section of the manuscript.**	Due to high levels of illiteracy consent was obtained verbally. Ethics approval from the Official University of Bukavu (UOB/CEM/007/2022) and New York University Abu Dhabi (HRPP-2022-34).

Powered by Editorial Manager® and ProduXion Manager® from Aries Systems Corporation

**Format for specific study types**<br><br>**Human Subject Research (involving human participants and/or tissue)**<br>• Give the name of the institutional review board or ethics committee that approved the study<br>• Include the approval number and/or a statement indicating approval of this research<br>• Indicate the form of consent obtained (written/oral) or the reason that consent was not obtained (e.g. the data were analyzed anonymously)<br><br>**Animal Research (involving vertebrate animals, embryos or tissues)**<br>• Provide the name of the Institutional Animal Care and Use Committee (IACUC) or other relevant ethics board that reviewed the study protocol, and indicate whether they approved this research or granted a formal waiver of ethical approval<br>• Include an approval number if one was obtained<br>• If the study involved *non-human primates*, add *additional details* about animal welfare and steps taken to ameliorate suffering<br>• If anesthesia, euthanasia, or any kind of animal sacrifice is part of the study, include briefly which substances and/or methods were applied<br><br>**Field Research**<br><br>Include the following details if this study involves the collection of plant, animal, or other materials from a natural setting:<br>• Field permit number<br>• Name of the institution or relevant body that granted permission	
**Data Availability**<br><br>Authors are required to make all data underlying the findings described fully available, without restriction, and from the time of publication. PLOS allows rare exceptions to address legal and ethical concerns. See the PLOS Data Policy and FAQ for detailed information.	Yes - all data are fully available without restriction

A Data Availability Statement describing where the data can be found is required at submission. Your answers to this question constitute the Data Availability Statement and **will be published in the article**, if accepted.<br><br>**Important:** Stating 'data available on request from the author' is not sufficient. If your data are only available upon request, select 'No' for the first question and explain your exceptional situation in the text box.<br><br>Do the authors confirm that all data underlying the findings described in their manuscript are fully available without restriction?	
**Describe where the data may be found in full sentences. If you are copying our sample text, replace any instances of XXX with the appropriate details.**<br><br>• If the data are **held or will be held in a public repository**, include URLs, accession numbers or DOIs. If this information will only be available after acceptance, indicate this by ticking the box below. For example: *All XXX files are available from the XXX database (accession number(s) XXX, XXX.).*<br>• If the data are all contained **within the manuscript and/or Supporting Information files**, enter the following: *All relevant data are within the manuscript and its Supporting Information files.*<br>• If neither of these applies but you are able to provide **details of access elsewhere**, with or without limitations, please do so. For example:<br><br>*Data cannot be shared publicly because of [XXX]. Data are available from the XXX Institutional Data Access / Ethics Committee (contact via XXX) for researchers who meet the criteria for access to confidential data.*<br><br>*The data underlying the results presented in the study are available from (include the name of the third party*	All data and replication files are available from Dataverse.

and contact information or URL).<br>• This text is appropriate if the data are owned by a third party and authors do not have permission to share the data.<br><br>* typeset	
Additional data availability information:	Tick here if the URLs/accession numbers/DOIs will be available only after acceptance of the manuscript for publication so that we can ensure their inclusion before publication.

Powered by Editorial Manager® and ProduXion Manager® from Aries Systems Corporation

Manuscript Click here to access/download;Manuscript;Manuscript.pdf

Where to flee? Preferences for host communities among displaced people in Congo

Nik Stoop[1¶], Peter van der Windt[2¶*], Sigrid Weber[3¶]

[1]Institute of Development Policy, University of Antwerp, Antwerp, Belgium

[2]Division of Social Science, New York University Abu Dhabi, Abu Dhabi, United Arab Emirates

[3]Immigration Policy Lab, Stanford University, Stanford, California, United States of America

¶These authors contributed equally to this work.

*Corresponding author: petervanderwindt@nyu.edu

Abstract

Previous research has explored host communities' attitudes toward displaced individuals, but we know much less about what displaced people seek in a host community. Understanding both perspectives is key to fostering successful local integration. This study responds to this gap through a conjoint experiment that randomly varies the characteristics of the host community. The experiment is embedded in a survey of nearly 2,000 respondents in the Kasai region of the Democratic Republic of Congo, an area heavily affected by conflict-induced displacement. Our findings indicate that in addition to safety, displaced individuals prioritize job opportunities but also desire access to a social and political life. Our results suggest that the promotion of economic opportunities and inclusive governance are among the most effective strategies to create conditions conducive to local integration.

Introduction

Forced displacement is a pressing global challenge. At the end of 2023, 117.3 million people were displaced due to conflicts or natural disasters [1]. Policymakers propose three durable "solutions" to the displacement challenge: resettlement in a third country, voluntary repatriation, and integration within host communities [2]. Resettlement, however, remains unattainable for most displaced individuals, and returning home is often impossible due to the persistent nature of conflict [1]. With global violence on the rise [3], local integration within host communities is becoming an increasingly important pathway for displaced individuals seeking opportunities to rebuild their lives.

Successful integration depends on aligning the needs of displaced individuals with the capacities and priorities of the host communities [4]. Existing research on displaced-host interactions largely emphasizes the preferences of host communities toward migrants (see, e.g., [5] and [6] for recent meta-analyses). Considerably less attention has been paid to the preferences of those displaced. Given that displaced people actively make movement decisions and exercise agency in choosing host communities [7, 8], understanding their preferences is equally vital to promote integration. Recent studies have begun to examine the movement and destination preferences of displaced populations [e.g., 9, 10, 11], while others have developed mechanisms to match refugees with host communities based on expected integration outcomes and settlement preferences [e.g., 12, 13].

We contribute to this emerging body of research by conducting a conjoint experiment in which we manipulate the characteristics of hypothetical host communities. This design allows us to assess the impact of host community characteristics on individuals' preferences for these host communities. Our research takes place in the Kasai provinces of the Democratic Republic of Congo (DRC), a country that is currently home to the world's third largest population of internally displaced people (IDPs) [14]. Between 2016 and 2017, the Kasai region experienced intense conflict, marked by the displacement of 1.4 million people [15].

Although active fighting decreased after 2017, pockets of insecurity remain, occasionally triggering further displacement [16, 17]. At the time of our data collection, in 2022-2023, hundreds of thousands of people remained displaced, and the region continued to struggle with high levels of poverty and food insecurity [18].

In collaboration with UNHCR, the UN Refugee Agency, we conducted a large-scale survey among people displaced by the conflict and members of the host community. We conducted a conjoint experiment with almost 2,000 respondents to learn about preferences for host communities that go beyond safety, focusing on factors related to job opportunities, church membership, family and ethnic connections, and participation in local decision-making. The findings reveal that respondents prioritize self-reliance, with a stronger preference for securing employment over other factors such as the presence of co-ethnics in the community. At the same time, they seek an active social and political life by participating in the local church community and local political decision-making.

These findings have important policy implications. Although certain community characteristics, such as family or ethnic ties, are fixed, others can be actively leveraged to support the integration of displaced populations. Exploring perceived integration mechanisms, our findings suggest that creating employment opportunities and stimulating participation in local decision making are among the most effective strategies. These factors not only enhance individuals' sense of economic contribution and agency, but also foster feelings of being welcomed, safe, and trusting towards the host community. By prioritizing these adaptable factors, policymakers and humanitarian organizations can create more conducive conditions for successful local integration.

Preferences for Host Communities

Although preferences for host communities are understudied, the broader phenomena of displacement flows and destination choice have received considerable attention. Most promi-

nently, conflict and violence have been found to shape when populations flee, when they return, and where they go (e.g. [19, 20, 21]). However, this body of literature also suggests a role for other factors in destination choice. We discuss these below and use them as the theoretical foundation to analyze individual preferences for host communities. The hypotheses that we put forward in this section were pre-specified in a pre-registered analysis plan (see OSF registry [redacted for anonymity]).

A prominent factor in this literature is related to economic opportunities. Refugees tend to move to economically prosperous countries [22], arguably due to their more generous welfare systems, and because the combination of low unemployment and high economic growth makes it easier to find a job [23]. Similar findings come from work on IDPs, who tend to move from remote and less developed areas to more industrial areas and cities within the same country [24, 25]. Based on these findings, we hypothesize that displaced individuals prefer host communities where they can earn a living.

Scholars have also studied the role of social factors, because these may decrease transition and adaptation costs. For example, refugee movements have been explained by colonial ties [26] and ethnic links [27] with the destination country. Furthermore, the presence of existing migrant communities in their home country has been found to increase the appeal of a country to asylum seekers [23]. Furthermore, at a more local level, displaced individuals tend to move to places where people share their ethnic and political identity [28, 29]. This study therefore also explores whether people prefer host communities in which they have social, relational, and cultural ties.

Finally, political factors may be important. Electoral outcomes in destination countries have been found to influence the share of refugees who apply for asylum, with a higher share of votes for right-wing populist parties correlating with a decrease in asylum applications [23]. At the community level, examples of development programs in Lebanon, Jordan, and the DRC further suggest that joint decision-making between displaced and host communities may promote social cohesion [4]. We therefore assess if host communities that provide space

to co-shape political decisions within the community are deemed more attractive.

Context and Research Design

Conflict and Displacement in the Greater Kasai

We set out to test these hypotheses in the context of the Kasai, Kasai-Central, and Kasai-Oriental provinces in the Democratic Republic of Congo (panel (a) in Figure 1). This region was the epicenter of the 2016-2017 Kasai conflict, which displaced over 1.4 million people within the DRC and forced approximately 35,000 refugees to seek safety in Angola [30]. Most IDPs did not seek safety in camps or cities, but sought refuge with host families in rural settlements [31]. Today, large parts of the Kasai population remain displaced, and the region is characterized by food insecurity, ethnic tensions, and occasional instances of violence (e.g., [18, 32]). A more detailed account of the history of the conflict can be found in Appendix S1.

Fig 1. Map of research area. Notes: Authors' rendering. Panel (a): Congo and the three Kasai provinces where the study took place. Panel (b): The 62 health zones with the 27 selected health zones. Panel (c): The 119 survey locations.

Data and Sample

In collaboration with UNHCR, we conducted a survey representing both individuals displaced during the Kasai conflict and members of host communities (defined as those without a history of displacement). First, we selected the 27 health zones in the Kasai provinces most affected by displacement (panel (b) in Figure 1). Since there is no complete and up-to-date list of villages and towns in the Kasai region, we made use of Google AI satellite imagery that maps dwellings (these data are freely available online: `https://sites.research.google/open-buildings`). We defined 'localities' as geographical units of one square kilometer that

contained at least 100 dwellings. Of the 5,302 identified localities, we randomly selected our study localities proportional to the number of dwellings in the health zones and localities. Next, we conducted a listing survey of all households in 119 selected localities (panel (c) in Figure 1) between July and November 2022. From a total of 33,500 households, we randomly selected 4,069 and collected survey data from them between 24 November 2022 and 16 February 2023. Approximately half of them (n= 1,965) were assigned to participate in the joint experiment that we analyze below. A detailed discussion of the sampling procedure can be found in Appendix S2. Voluntary and informed consent was obtained from all participants. Consent was provided verbally due to high levels of illiteracy. We further discuss the ethics of conducting surveys in a humanitarian context in Appendix S2.

The final sample for the conjoint experiment consists of individuals who were forced to flee during the Kasai conflict (74%), and those who did not (26%). Half of the respondents are men (50%). The typical respondent is married (73%), has attended (88%) and graduated from primary school (74%), and can read (77%). Approximately 50% of the respondents did not have work in the week prior to the survey and 85% report not having a regular job. 12% of the respondents report experiences of violence in the past year. The definitions and summary information for all variables in this study can be found in Appendix S3.

Conjoint Experiment

We used a conjoint experiment to measure individual preferences for host community characteristics. As part of the survey, respondents were presented with two potential host communities that randomly varied across five attributes: job availability (to measure the economic dimension of integration), church membership (social integration), family ties (relational integration), language ties (cultural integration), and the ability to participate in village decisions (political integration). Table 1 provides an overview of the attributes and their levels, represented by drawings made by a Congolese artist. The experiment's attributes, corresponding levels, and the drawings were carefully piloted, and adjusted, between June

and October 2022; i.e., the five months preceding the survey.) When presented with the two communities, respondents were asked to imagine having recently fled violence and to choose which of the two safe host communities they would prefer to live in. In this experiment we thus take it as given that the potential host communities are safe. Conditional on safety, displaced people have a choice among different hosting communities and face trade-offs in terms of economic, social, cultural or political integration. Participants completed two rounds of the conjoint experiment, involving a total of 3,930 forced choices (i.e. 7,860 observations). To avoid ordering effects, the order of attributes was randomized between respondents, but fixed across rounds for the same respondent.

Table 1: Conjoint Experiment Attributes and Levels

Economic integration

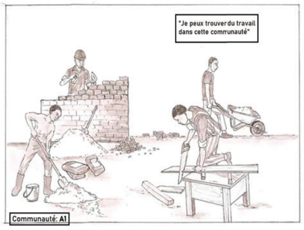

"You can get work in this village"

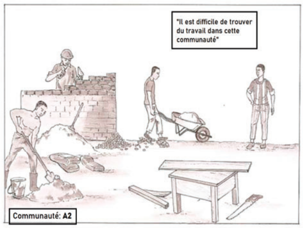

"It is difficult to get work in this village"

Social integration

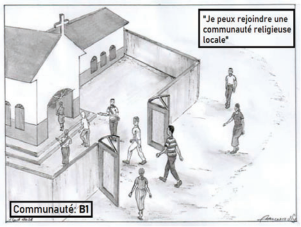

"You can join the local church"

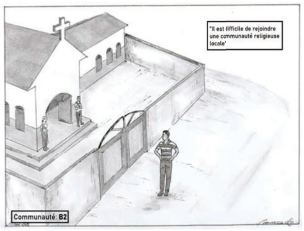

"You cannot join the local church"

Relational integration

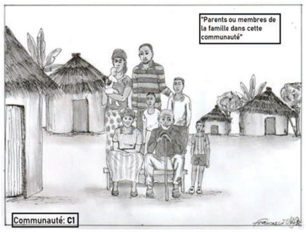

"You have relatives in the community"

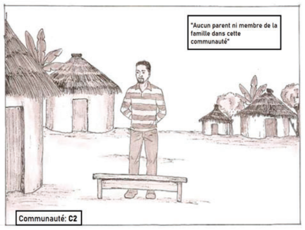

"You do not have relatives in the community"

Cultural integration

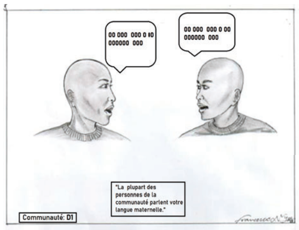

"The majority of the community speaks your mother tongue"

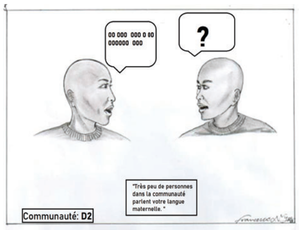

"Few people in the community speak your mother tongue"

Political integration

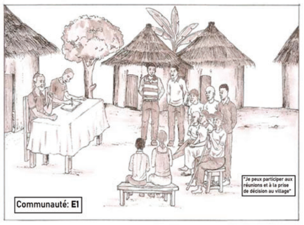

"You can attend village level meetings and participate in decision-making"

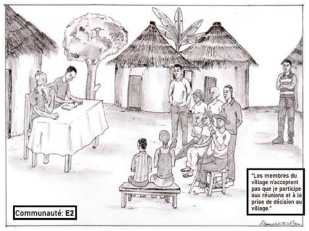

"Village members do not want you to come to village meetings"

Notes: Drawings conjoint. Drawings created by a Congolese artist. The experiment's attributes, corresponding levels, and the drawings were carefully piloted, and adjusted, between June and October 2022; i.e., the five months preceding the survey.

Results

Which Host Communities are Preferred?

Figure 2 presents Average Marginal Component Effects (AMCE), showing the causal impact of each attribute on a respondent's host community choice [33]. Appendix S4 presents estimates using marginal means [34], and AMCEs for a continuous outcome measure where we ask respondents to rank how much they would like to live in each of the host communities. Results are similar. Dots are the AMCE point estimates and thin (thick) horizontal lines show 95% confidence intervals. Securing a livelihood in the host community is the most important consideration. The probability of selecting a host community increases by 10.5 percentage points (pp, $p<0.01$) when work opportunities are available compared to when they are not. Social and cultural factors are also important: being able to join a local church and having relatives and co-ethnics in the community all significantly increase the attractiveness of host communities (by 6.8, 5.9 and 4.1 pp, $p<0.01$, respectively). Finally, respondents show a preference for political participation in the host community. The ability to attend village meetings and participate in local decision making increases the likelihood of choosing a host community by 5.2pp ($p<0.01$).

Fig 2. Preferences for Host Community (AMCE). Notes: Average Marginal Component Effects for Host Community Choice (N = 7,860). Dots are the AMCE point estimates and thin (thick) horizontal lines show 95% (90%) confidence intervals. Full numeric results can be found in Table S6.

Why are These Host Communities Preferred?

What mechanisms drive the effect of these host community attributes on individual preferences? The survey was designed to investigate this question. After making their choice,

respondents assessed five statements for each host community presented to them: i) I could contribute to the economy of this community, ii) I would feel welcomed in this community, iii) I would feel safe in this community, iv) I could trust the members of this community, and v) I could improve this community by bringing in new ideas and cultures. Responses are on a five-point Likert scale: not at all, not really, neither agree nor disagree, somewhat, strongly agree.

Figure 3 shows that respondents see economic and political integration as two distinct and important features of life in a host community. Respondents are considerably more likely to see themselves as contributing to the economy in communities with job opportunities, increasing their perceived ability to contribute by 0.8 on a five-point scale ($p<0.01$). The ability to participate in local decision making increases the perceived likelihood of contributing ideas to the community (0.7, $p<0.01$). Economic and political participation thus do not seem to support each other in a strongly reinforcing way. Both attributes, however, also have a positive impact on respondents feeling more welcome, safe, and trusting toward the receiving community. In contrast, host community attributes related to social or cultural factors, such as joining a local church, having relatives in the community, and speaking the same language, have a less pronounced impact on how respondents perceive integration in a host community.

Fig 3. Effect of Community Attributes on Perceived Integration Mechanisms (AMCE). Notes: Average marginal component effects for integration statements (N = 7,860). Dots are the AMCE point estimates and thin (thick) horizontal lines show 95% (90%) confidence intervals. Full numeric results can be found in Table S7.

Preferences by Displacement Experience

By design, our sample included individuals who had been displaced during the Kasai conflict, as well as those who had not (members of the host community). Figure S1 in the appendix

explains how these groups were defined. We investigate whether these groups hold different preferences for host communities. Figure 4 shows that although individuals displaced during the Kasai conflict generally exhibit slightly stronger preferences across all attributes, particularly for employment opportunities, the differences in AMCEs are not statistically significant. One possible explanation for this finding is that households in the Kasai provinces endure harsh living conditions regardless of whether they have experienced displacement. Indeed, displaced and non-displaced households in our sample are very similar across a wide set of key indicators, such as employment, food security, family structure, health and others (Table S11 in the appendix). Another possible explanation is that, as part of the the conjoint experiment, respondents were asked to imagine having recently fled violence. Members of the host community, who also faced considerable violence during the Kasai conflict, may be equally able to put themselves in the shoes of those who fled violence.

Fig 4. AMCE for Host Community Preferences by Displacement Experience. Notes: Average Marginal Component Effects for Host Community Choice (N = 7,860) by displacement experience. Dots are the AMCE point estimates and thin (thick) horizontal lines show 95% (90%) confidence intervals. Full numeric results can be found in Table S8.

Conclusion

Integration within host communities is an increasingly important pathway for displaced individuals to rebuild their lives [2]. Although much research has investigated the preferences of host communities toward migrants, much less attention has been paid to the characteristics that displaced individuals consider important in a host community. In this study, we partnered with UNHCR, the UN Refugee Agency, and conducted a conjoint experiment to respond to this gap in the literature. The experiment was embedded in a large-scale survey conducted in the Kasai provinces of the Democratic Republic of Congo.

Our findings indicate that while displaced individuals prioritize securing a livelihood,

they also care about building a social and political life. As such, they significantly prefer host communities that facilitate social integration - by joining the local church or through the presence of relatives and co-ethnics - and those that allow them to participate in local decision-making. The results suggest that fostering employment opportunities and encouraging participation in local decision making are key strategies for policymakers and humanitarian organizations to support the local integration of displaced individuals. These factors enhance individuals' sense of economic contribution and agency, while also promoting feelings of being welcomed, safe, and trusting towards the host community. The similarity in preferences among those respondents with and without a displacement experience suggests that these factors resonate with broader societal expectations.

We consider this study a first step and encourage future research in two directions: i) exploring the role of other host community attributes and different contexts in shaping host community preferences, and ii) better understanding the constraints that displaced populations operate under that may generate location choices differing from their location preferences. Related to the latter, whereas in developed countries there may be relatively little agency by displaced individuals in their host community choice (often refugees are assigned to host locations by government agencies (e.g., Jones and Teytelboym 13), in the developing world location choice decisions are often a function of an individual's informational, financial, and logistical constraints. Measuring preferences for host community characteristics and actual host community characteristics, and obtaining a better understanding about why they may differ is crucial to improve our understanding of integration dynamics.

Given the already large scale of forced displacement and the expectation of further escalation due to ongoing violence, desertification, rising sea levels, and more frequent severe weather events, it is of central importance to gain a better understanding of the determinants for successful integration. The preferences of displacement populations for the characteristics of the host community are one piece of this puzzle.

Acknowledgments

We thank Mateo Villamizar Chaparro, Jens Hainmueller, J. Andrew Harris, Oguzhan Türkoglu, Jeremy Weinstein, Mark Marvin Kadigo, and participant at Stanford's Immigration Policy Lab and at the Violence, Instability, and Peace Workshop for comments. Thanks also to Victor Daye, Marguerite Duponchel, Mamadou Fofana, Emmanuel Kandate, Hyacine Manawa, Harriet Kasidi Mugera, Papa Moussa Ndoye, Sergiy Radyakin, and Michael Wild. We thank Congo's National Institute of Statistics and the surveyors for data collection. We thank the World Bank-UNHCR Joint Data Center on Forced Displacement and New York Abu Dhabi for funding. We would like to thank Francois Nyamo for the artwork used in this study. Ethics approval from the Official University of Bukavu (UOB/CEM/007/2022) and New York University Abu Dhabi (HRPP-2022-34).

References

[1] UNHCR. Global Trends: Forced Displacement in 2023, 2024. URL `https://www.unhcr.org/global-trends-report-2023`.

[2] UNHCR. Solutions, 2025. URL `https://www.unhcr.org/what-we-do/build-better-futures/solutions`. Accessed: 2025-03-26.

[3] Siri Aas Rustad. Conflict trends: A global overview: 1946-2023, 2024. URL `https://www.prio.org/publications/14006`.

[4] World Bank. Social cohesion and forced displacement: A synthesis of new research. Technical report, 2022. URL `www.worldbank.org`.

[5] Sigrid Weber, Nik Stoop, Peter Van der Windt, and Haoyu Zhai. A meta-analysis of attitudes towards migrants and displaced persons. *Stanford IPL Working Paper Series*, 2024.

[6] Marco Aviña, Taeku Lee, Mashail Malik, Reed Rasband, Marcel Roman, and Priyanka Sethy. Which immigrants do citizens prefer? a meta-reanalysis of 96 conjoint experiments. Unpublished working paper, 2024.

[7] Sinem Kavak, Mo Hamza, Thomas Gammeltoft-Hansen, and Russell A. Stone. Refugee agency in secondary mobility decision-making: A systematic literature review. *Frontiers in Human Dynamics*, 6, 2024. doi: 10.3389/fhumd.2024.1376968.

[8] Oleksandra Tarkhanova and Daryna Pyrogova. Forced displacement in ukraine: Understanding the decision-making process. *European Societies*, 26(2):481–500, 2024. doi: 10.1080/14616696.2023.2280680.

[9] Faten Ghosn, Tiffany S. Chu, Miranda Simon, Alex Braithwaite, Michael Frith, and Joanna Jandali. The journey home: Violence, anchoring, and refugee deci-

sions to return. *American Political Science Review*, 115(3):982–998, 2021. doi: 10.1017/S0003055421000344.

[10] Ahmet Akbiyik, Karen E. Ferree, and Kristen Kao. Preferences for segregation? a study of migrants and hosts in adana, turkey, Sep 2024. Available at SSRN: `https://ssrn.com/abstract=4957757` or `http://dx.doi.org/10.2139/ssrn.4957757`.

[11] Mikael Elinder, Oscar Erixson, and Olle Hammar. Where would ukrainian refugees go if they could go anywhere? *International Migration Review*, 57:587–602, 6 2023.

[12] Avidit Acharya, Kirk Bansak, and Jens Hainmueller. Combining outcome-based and preference-based matching: A constrained priority mechanism. *Political Analysis*, 30: 89–112, 2022.

[13] Will Jones and Alexander Teytelboym. The local refugee match: Aligning refugees' preferences with the capacities and priorities of localities. *Journal of Refugee Studies*, 31:152–178, 6 2018.

[14] IDMC. Global report on internal displacement 2023. Technical report, 2023.

[15] Mercy Corps. Kasai conflict assessment. Technical report, Mercy Corps, 2019.

[16] UNHCR. Resurging violence could spark mass displacement in drc's kasai, unhcr warns, 2020.

[17] UNHCR. Violent attacks displace thousands in dr congo's kasai region, 2021.

[18] IPC. Ipc acute food insecurity snapshot. Technical report, 2023.

[19] Christian A. Davenport, Will H. Moore, and Steven C. Poe. Sometimes you just have to leave: Domestic threats and forced migration: 1964-1989. *International Interactions*, 29:27–55, 2003. doi: 10.1080/03050620390182966.

[20] Ala Alrababah, Daniel Masterson, Marine Casalis, Dominik Hangartner, and Jeremy Weinstein. The dynamics of refugee return: Syrian refugees and their migration intentions. *British Journal of Political Science*, 53:1108–1131, 2023. ISSN 14692112.

[21] Oguzhan Turkoglu and Sigrid Weber. When to go? a conjoint experiment on social networks, violence, and forced migration decisions in eastern and southeastern turkey. *International Studies Quarterly*, 67, 6 2023.

[22] Marie McAuliffe and Dinuk Jayasuriya. Do asylum seekers and refugees choose destination countries? evidence from large-scale surveys in australia, afghanistan, bangladesh, pakistan and sri lanka. *International Migration*, 54:44–59, 8 2016. ISSN 14682435. doi: 10.1111/imig.12240.

[23] Eric Neumayer. Asylum destination choice: What makes some west european countries more attractive than others? *European Union Politics*, 5:155–180, 2004.

[24] Mathias Czaika and Krisztina Kis-Katos. Civil conflict and displacement: Village-level determinants of forced migration in aceh. *Journal of Peace Research*, 46:399–418, 2009.

[25] Stefanie Engel and Ana María. Displacement due to violence in colombia: A household-level analysis. *Economic Development and Cultural Change*, 55:335–365, 2007.

[26] Will H Moore and Stephen M Shellman. Whither will they go? a global study of refugees' destinations, 1965-1995. *International Studies Quarterly*, 51:811–834, 2007.

[27] Seraina Rüegger and Heidrun Bohnet. The ethnicity of refugees (er): A new dataset for understanding flight patterns. *Conflict Management and Peace Science*, 35:65–88, 1 2018. ISSN 15499219. doi: 10.1177/0738894215611865.

[28] Abbey Steele. Seeking safety: Avoiding displacement and choosing destinations in civil wars. *Journal of Peace Research*, 46:419–430, 2009.

[29] Laia Balcells. Dynamics of internal resettlement during civil war. *Journal of Peace Research*, 55:236–251, 2018. doi: 10.2307/48596168.

[30] UNHCR. Update on unhcr's operations in africa. Technical report, 2017.

[31] Oxfam. Kasai: The forgotten Province of DRC. Technical report, Oxfam, 2017.

[32] Radio Okapi. Kasai-central: plus de 100 ménages déplacés et des morts à la suite des affrontements communautaires à dimbelenge. Technical report, Radio Okapi, 2023.

[33] Jens Hainmueller, Daniel J Hopkins, and Teppei Yamamoto. Causal inference in conjoint analysis: Understanding multidimensional choices via stated preference experiments. *Political Analysis*, 22(1):1–30, 2014.

[34] Thomas J. Leeper, Sara B. Hobolt, and James Tilley. Measuring subgroup preferences in conjoint experiments. *Political Analysis*, 28(2):207–221, 2020. doi: 10.1017/pan.2019.30.

Figure 1a

Click here to access/download;Figure;Fig1a.tiff

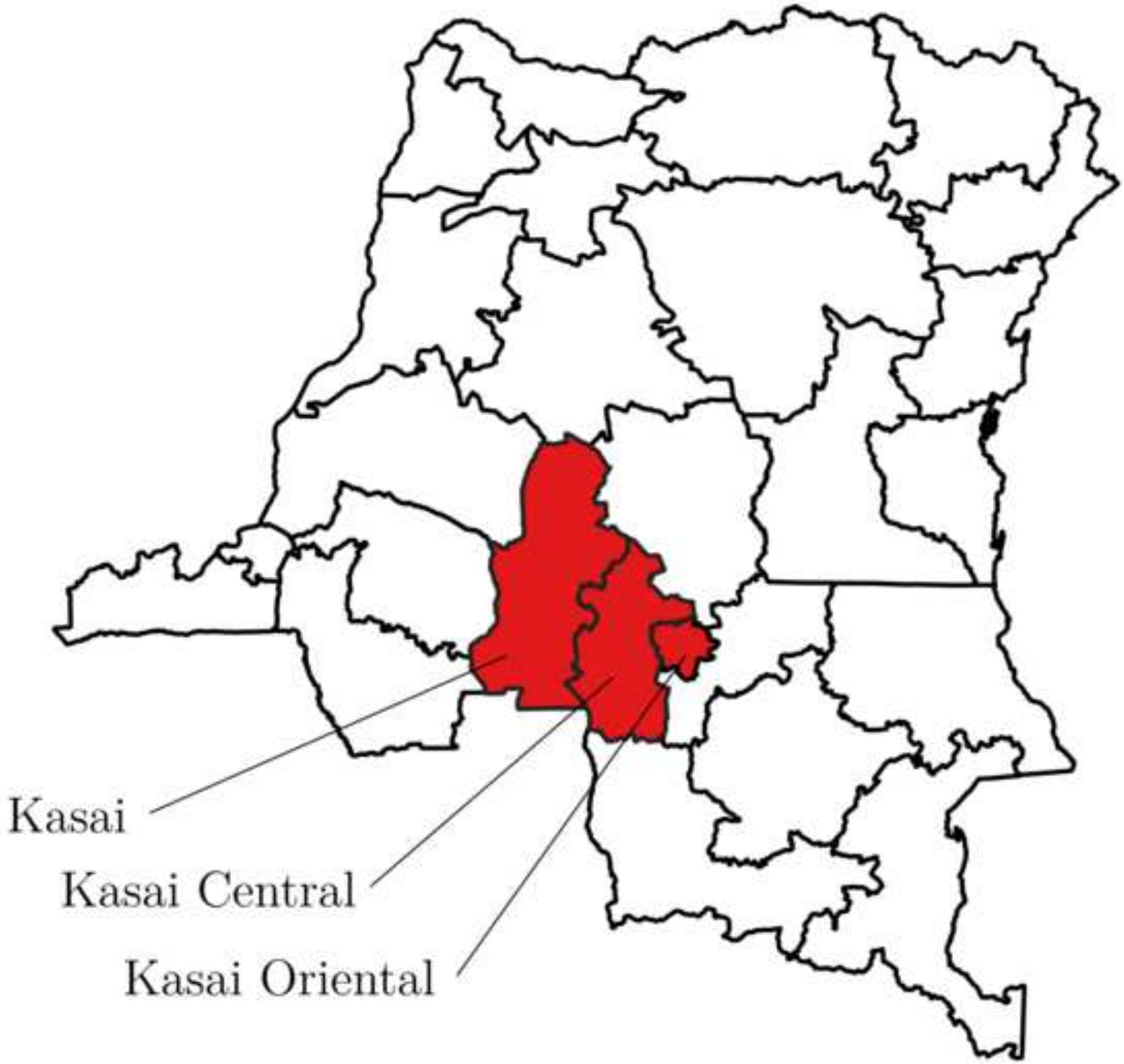

Figure 1b

Click here to access/download;Figure;Fig1b.tiff

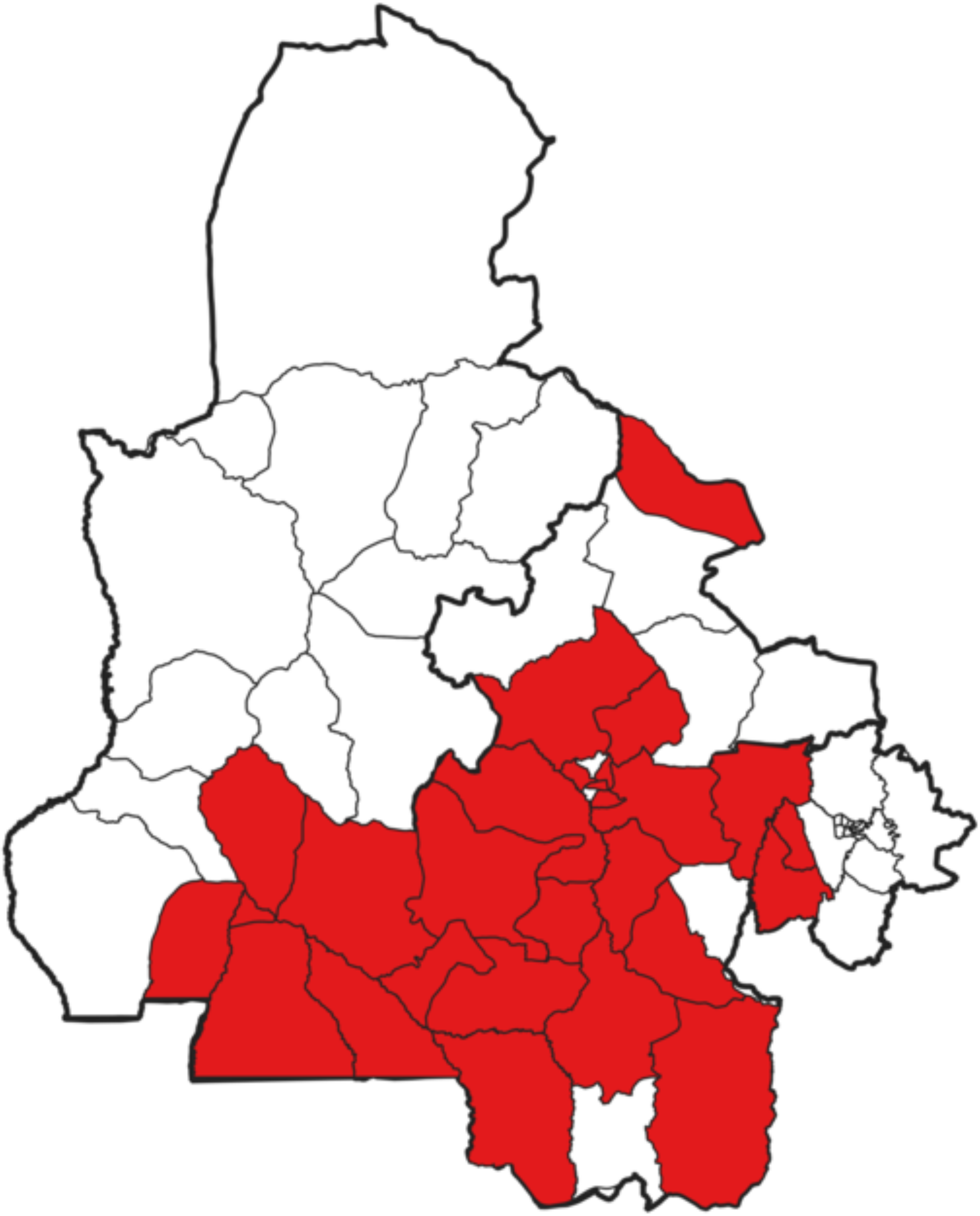

Figure 1c

Click here to access/download;Figure;Fig1c.tiff

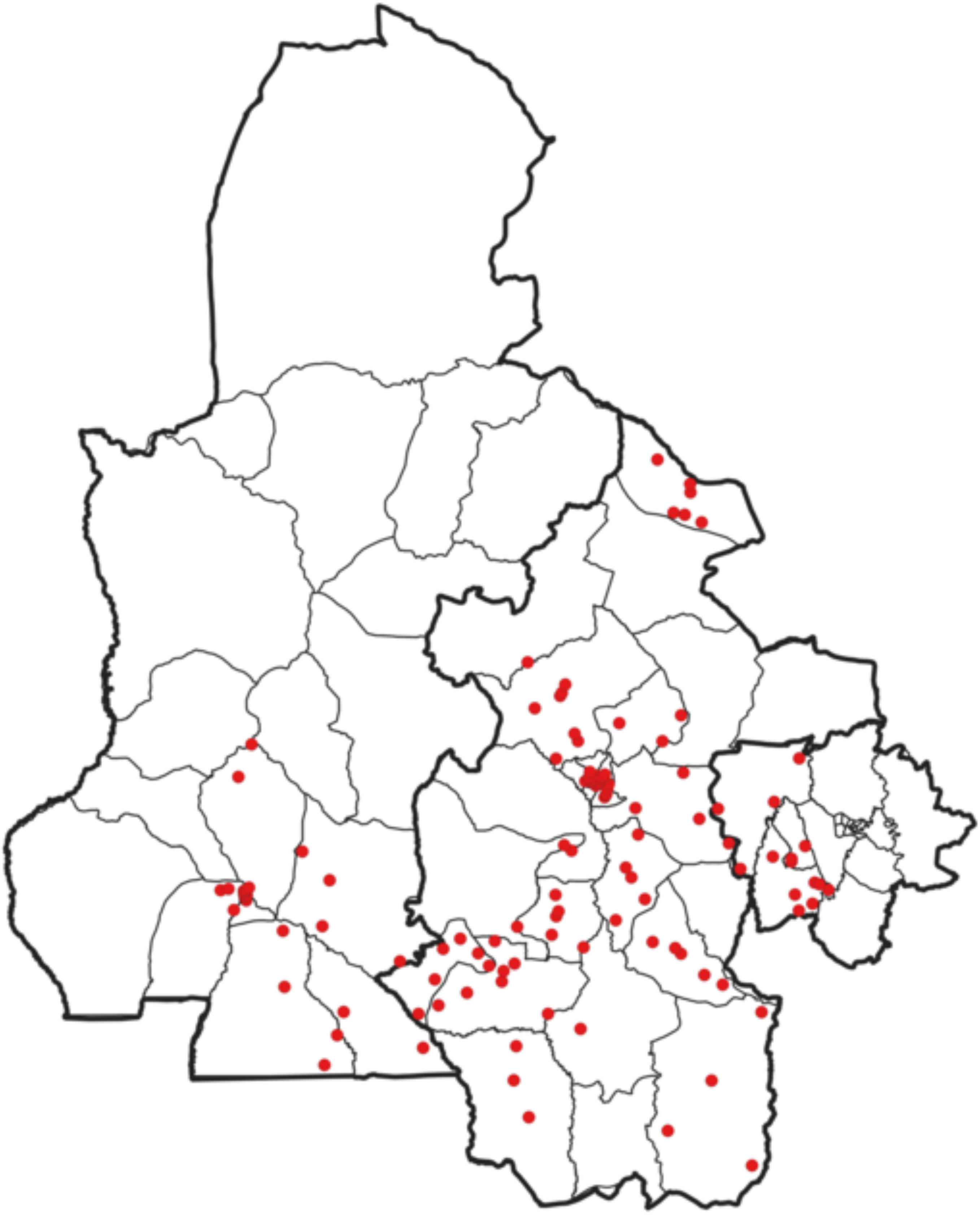

Figure 2

Click here to access/download;Figure;Fig2.tiff

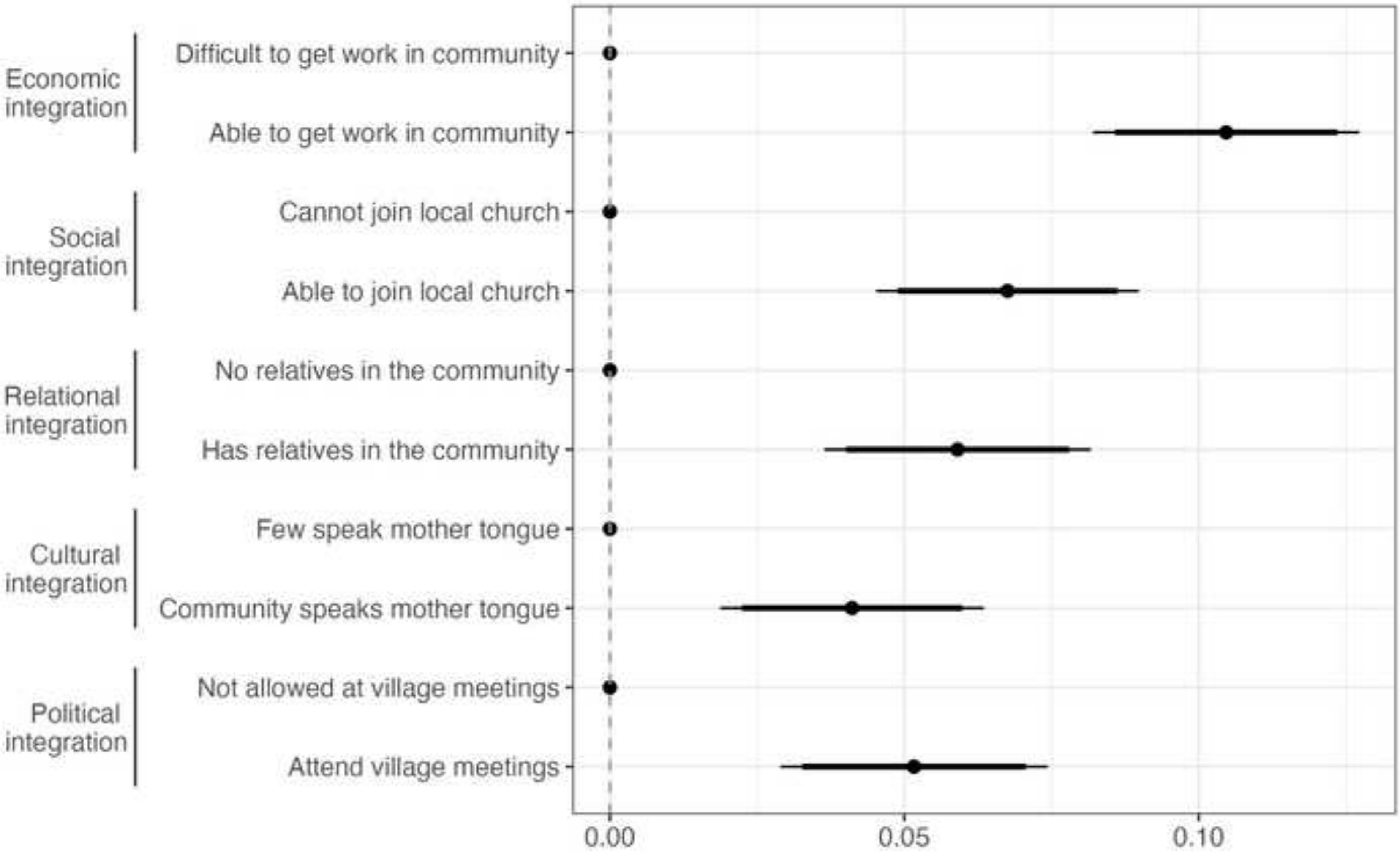

Figure 3

Click here to access/download;Figure;Fig3.tiff

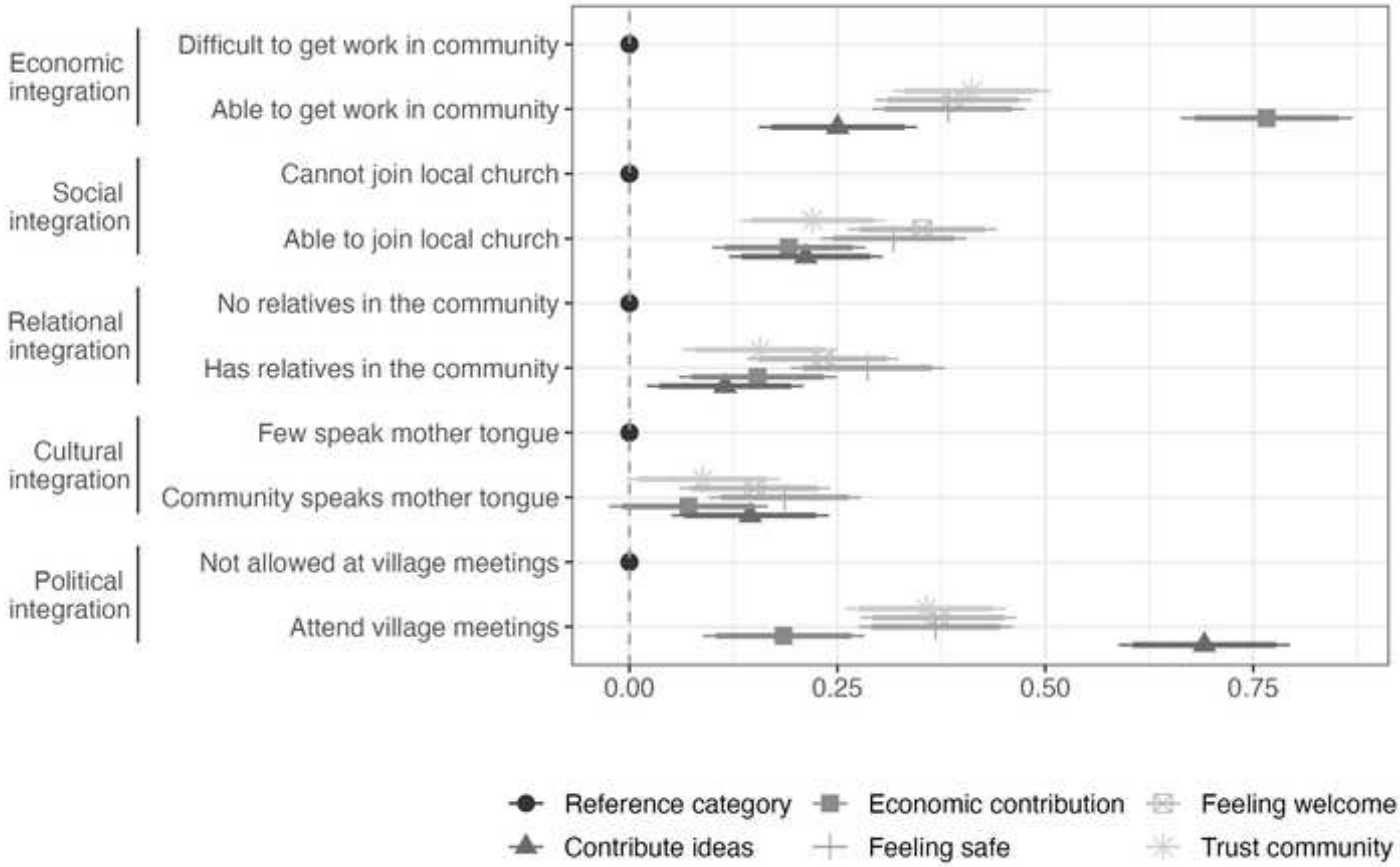

Figure 4

Click here to access/download;Figure;Fig4.tiff

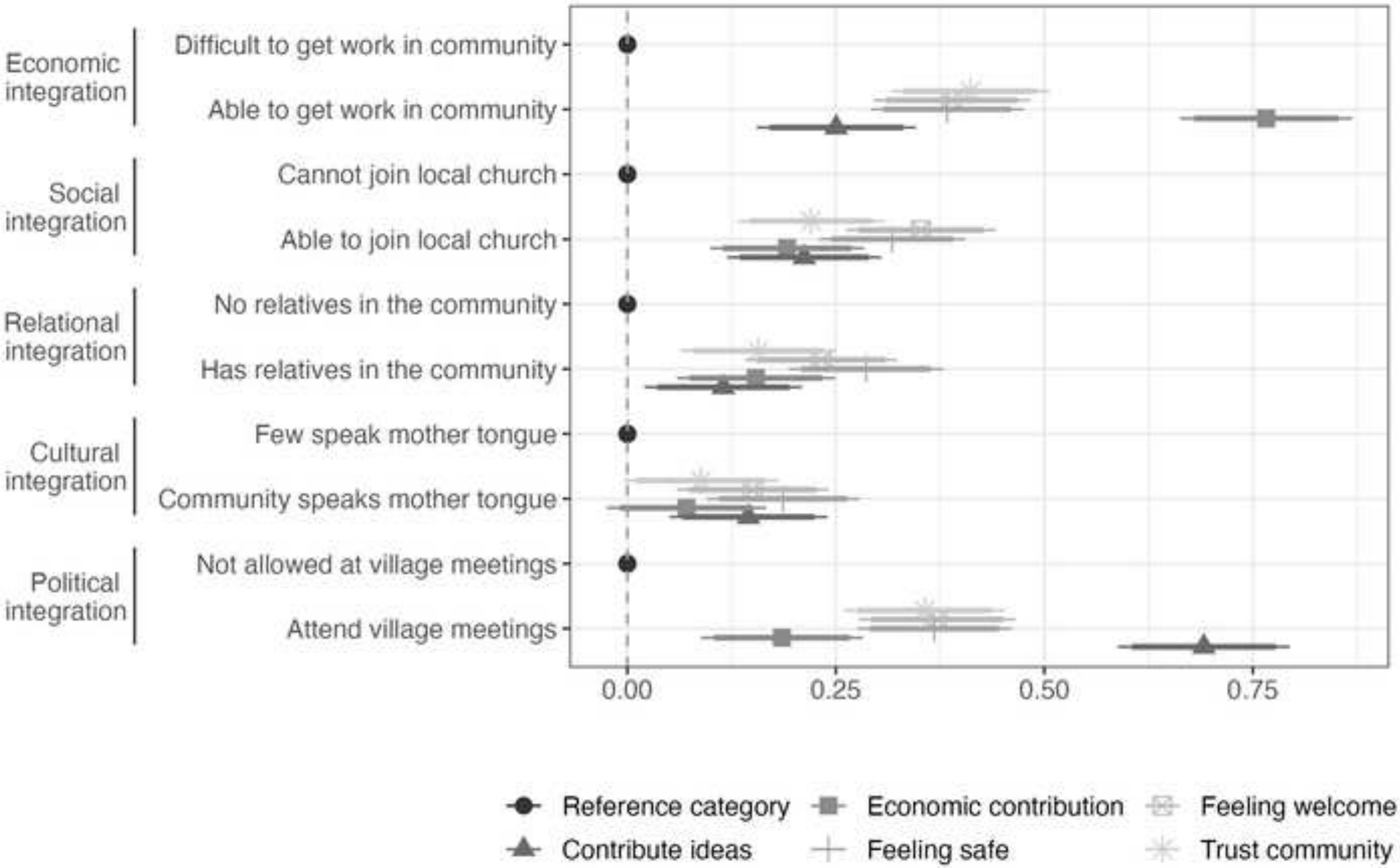

Click here to access/download
Supporting Information
Supporting Information.pdf
