# Supplementary material for: Where to flee? Preferences for host communities among displaced people in Congo
Source: PLoS One. 2025 Dec 9;20(12):e0337530. doi: 10.1371/journal.pone.0337530 (PMC12688085; doi:10.1371/journal.pone.0337530)
Supplement: Attachment — Submitted filename: Response Letter.pdf [file pone.0337530.s003.pdf]

September 29, 2025

Subject: Response to Reviewers

Dear Professor Parker,

Thank you for the opportunity to revise the paper. We thank you and the Reviewers for their constructive comments, which helped make the paper considerably stronger. Here is a brief summary of the major changes:

- We no longer only separate out displaced and non-displaced populations but explicitly discuss all subgroups in our sample: IDPs, returned IDPs, repatriated refugees and host community members. As part of this, we have edited the text throughout the manuscript, updated the related results figure, and provide summary information by subgroup.
- We have undertaken several additional analyses, including controlling for covariates and conducting subgroup analyses by i) respondent literacy status, and ii) respondent ability to speak the main local language.
- We have made edits throughout the manuscript to enhance clarity and flow. In particular, we are now more careful in using the term ethnicity.
- All data and instruments are now publicly available online. We refer to the public depositories in the main text.
- We also refer to the publicly available analysis plan, which we registered before data collection.
- We now comply with all journal requirements: we have written the manuscript in the PLOS ONE template, uploaded our figures to the PACE digital diagnostic tool, etc.

Below we first address the issues you raised regarding journal requirements. Next, we address each of the Reviewers' comments individually.

In addition to this response letter, we have included the following items:

- A cover letter with an updated funding statement because we made changes to the financial disclosure as you suggested following the journal requirements ("Cover Letter").
- A marked-up copy of the manuscript that highlights changes made to the original version ("Revised Manuscript with Track Changes")
- An unmarked version of our revised paper without tracked changes ("Manuscript")
- An unmarked version of the supplementary Information ("Supporting Information")
- A filled-out inclusivity in global research questionnaire ("Inclusivity-in-global-research-questionnaire")
- A completed Content Permission Form for the content of Table 1 ("content-permission-form Table1 signed")

Many thanks again to you and the Reviewers for your time and consideration. We remain at your disposal for any additional clarifications, corrections or additions.

The authors

Response to Journal Requirements

"1. Please ensure that your manuscript meets PLOS ONE's style requirements, including those for file naming. The PLOS ONE style templates can be found at

https://journals.plos.org/plosone/s/file?id=wjVg/PLOSOne_formatting_sample_main_body.pdf and https://journals.plos.org/plosone/s/file?id=ba62/PLOSOne_formatting_sample_title_authors_affiliations.pdf"

We have moved the manuscript to the PLOS ONE style template, and the manuscript now meets the style requirements.

"2. Please include a complete copy of PLOS' questionnaire on inclusivity in global research in your revised manuscript. Our policy for research in this area aims to improve transparency in the reporting of research performed outside of researchers' own country or community. The policy applies to researchers who have travelled to a different country to conduct research, research with Indigenous populations or their lands, and research on cultural artefacts. The questionnaire can also be requested at the journal's discretion for any other submissions, even if these conditions are not met. Please find more information on the policy and a link to download a blank copy of the questionnaire here: https://journals.plos.org/plosone/s/best-practices-in-research-reporting. Please upload a completed version of your questionnaire as Supporting Information when you resubmit your manuscript."

We have filled out the questionnaire on inclusivity in global research and have attached it to our re-submission. We have also included the suggested subsection "Inclusivity in global research" in the methods section.

"3. In the ethics statement in the Methods, you have specified that verbal consent was obtained. Please provide additional details regarding how this consent was documented and witnessed, and state whether this was approved by the IRB."

We have added our strategy to obtain verbal consent, which was indeed approved by the IRB, to the methods section.

"4. Please update your submission to use the PLOS LaTeX template. The template and more information on our requirements for LaTeX submissions can be found." at http://journals.plos.org/plosone/s/latex.

We have now written the manuscript using the PLOS LaTeX template.

"5. Please expand the acronym "UNHCR" (as indicated in your financial disclosure) so that it states the name of your funders in full.

This information should be included in your cover letter; we will change the online submission form on your behalf."

We have expanded the acronym UNHCR in the funding statement, and we have included this information in the updated cover letter.

"6. Thank you for stating the following in the Acknowledgments Section of your manuscript:

[We thank Mateo Villamizar Chaparro, Jens Hainmueller, J. Andrew Harris, Oguzhan T¨urkoglu,237 Jeremy Weinstein, Mark Marvin Kadigo, and participant at Stanford's Immigration Policy 238 Lab and at the Violence, Instability, and Peace Workshop for comments. Thanks also to 239 Victor Daye, Marguerite Duponchel, Mamadou Fofana, Emmanuel Kandate, Hyacine Man- 240 awa, Harriet Kasidi Mugera, Papa Moussa Ndoye, Sergiy Radyakin, and Michael Wild. We 241 thank Congo's National Institute of Statistics and the surveyors for data collection. We 242 thank the World Bank-UNHCR Joint Data Center on Forced Displacement and New York 243 Abu Dhabi for funding. We would like to thank Francois Nyamo for the artwork used in this 244 study. Ethics approval from the Official University of Bukavu (UOB/CEM/007/2022) and 245 New York University Abu Dhabi (HRPP-2022-34).]

We note that you have provided funding information that is currently declared in your Funding Statement. However, funding information should not appear in the Acknowledgments section or other areas of your manuscript. We will only publish funding information present in the Funding Statement section of the online submission form.

Please remove any funding-related text from the manuscript and let us know how you would like to update your Funding Statement. Currently, your Funding Statement reads as follows:

[NS and PW received funding from the World Bank UNHCR Joint Data Center. The funders did not play a role in the study design, data collection and analysis, decision to publish, or preparation of the manuscript]

Please include your amended statements within your cover letter; we will change the online submission form on your behalf."

We have removed funding-related text from the manuscript. In our cover letter, we have provided an updated text for the Funding Statement.

"7. When completing the data availability statement of the submission form, you indicated that you will make your data available on acceptance. We strongly recommend all authors decide on a data sharing plan before acceptance, as the process can be lengthy and hold up publication timelines. Please note that, though access restrictions are acceptable now, your entire data will need to be made freely accessible if your manuscript is accepted for publication. This policy applies to all data except where public deposition would breach compliance with the protocol approved by your research ethics board. If you are unable to adhere to our open data policy, please kindly revise your statement to explain your reasoning and we will seek the editor's input

on an exemption. Please be assured that, once you have provided your new statement, the assessment of your exemption will not hold up the peer review process."

All data, including survey instruments, have been anonymized and made freely accessible on the Harvard Dataverse website: https://dataverse.harvard.edu/dataset.xhtml?persistentId=doi:10.7910/DVN/PHKNSU. We have included this information in the manuscript (page 4).

"8. We note that you have referenced (Marco Avi˜na, Taeku Lee, Mashail Malik, Reed Rasband, Marcel Roman, and Priyanka Sethy. Which immigrants do citizens prefer? a meta-reanalysis of 96 conjoint experiments. Unpublished working paper, 2024) which has currently not yet been accepted for publication. Please remove this from your References and amend this to state in the body of your manuscript: (Marco Avi˜na, Taeku Lee, Mashail Malik, Reed Rasband, Marcel Roman, and Priyanka Sethy. Which immigrants do citizens prefer? a meta-reanalysis of 96 conjoint experiments. Unpublished working paper, 2024) as detailed online in our guide for authors: http://journals.plos.org/plosone/s/submission-guidelines#loc-reference-style"."

This paper is now available online as an OSF preprint (https://osf.io/preprints/osf/etzmj_v2). We have updated this reference.

"9. Your ethics statement should only appear in the Methods section of your manuscript. If your ethics statement is written in any section besides the Methods, please delete it from any other section."

As requested, the ethics statement now only appears in the Methods section. We initially also had a subsection "Ethical Considerations" in the Supplementary Information, which we have now incorporated into the main document's ethics statement in the Methods section.

"10. We note that Figures 1a, 1b, and 1c in your submission contain map images which may be copyrighted. All PLOS content is published under the Creative Commons Attribution License (CC BY 4.0), which means that the manuscript, images, and Supporting Information files will be freely available online, and any third party is permitted to access, download, copy, distribute, and use these materials in any way, even commercially, with proper attribution. For these reasons, we cannot publish previously copyrighted maps or satellite images created using proprietary data, such as Google software (Google Maps, Street View, and Earth). For more information, see our copyright guidelines: http://journals.plos.org/plosone/s/licenses-and-copyright.

We require you to either (1) present written permission from the copyright holder to publish these figures specifically under the CC BY 4.0 license, or (2) remove the figures from your submission:

1. You may seek permission from the original copyright holder of Figures 1a, 1b, and 1c to publish the content specifically under the CC BY 4.0 license.

We recommend that you contact the original copyright holder with the Content Permission Form (http://journals.plos.org/plosone/s/file?id=7c09/content-permission-form.pdf) and the following text:

"I request permission for the open-access journal PLOS ONE to publish XXX under the Creative Commons Attribution License (CCAL) CC BY 4.0 (http://creativecommons.org/licenses/by/4.0/). Please be aware that this license allows unrestricted use and distribution, even commercially, by third parties. Please reply and provide explicit written permission to publish XXX under a CC BY license and complete the attached form."

Please upload the completed Content Permission Form or other proof of granted permissions as an "Other" file with your submission.

In the figure caption of the copyrighted figure, please include the following text: "Reprinted from [ref] under a CC BY license, with permission from [name of publisher], original copyright [original copyright year]."

2. If you are unable to obtain permission from the original copyright holder to publish these figures under the CC BY 4.0 license or if the copyright holder's requirements are incompatible with the CC BY 4.0 license, please either i) remove the figure or ii) supply a replacement figure that complies with the CC BY 4.0 license. Please check copyright information on all replacement figures and update the figure caption with source information. If applicable, please specify in the figure caption text when a figure is similar but not identical to the original image and is therefore for illustrative purposes only.

The following resources for replacing copyrighted map figures may be helpful:

USGS National Map Viewer (public domain): http://viewer.nationalmap.gov/viewer/
The Gateway to Astronaut Photography of Earth (public domain): http://eol.jsc.nasa.gov/sseop/clickmap/
Maps at the CIA (public domain): https://www.cia.gov/library/publications/the-world-factbook/index.html and https://www.cia.gov/library/publications/cia-maps-publications/index.html
NASA Earth Observatory (public domain): http://earthobservatory.nasa.gov/
Landsat: http://landsat.visibleearth.nasa.gov/
USGS EROS (Earth Resources Observatory and Science (EROS) Center) (public domain): http://eros.usgs.gov/#
Natural Earth (public domain): http://www.naturalearthdata.com/"

We have created the subfigures for "Fig 1. Map of research area" in QGIS, relying on shapefiles that are publicly available from the Humanitarian Data Exchange (https://data.humdata.org).

To clarify this, we have now added the following text in the Figure's note: "Maps created by the authors in QGIS using public domain shapefiles from the Humanitarian Data Exchange (HDX, OCHA)."

"11. We note that Table 1 in your submission contains copyrighted images. All PLOS content is published under the Creative Commons Attribution License (CC BY 4.0), which means that the manuscript, images, and Supporting Information files will be freely available online, and any third party is permitted to access, download, copy, distribute, and use these materials in any way, even commercially, with proper attribution. For more information, see our copyright guidelines: http://journals.plos.org/plosone/s/licenses-and-copyright.

We require you to either (1) present written permission from the copyright holder to publish these figures specifically under the CC BY 4.0 license, or (2) remove the figures from your submission:

1. You may seek permission from the original copyright holder of Table 1 to publish the content specifically under the CC BY 4.0 license.

We recommend that you contact the original copyright holder with the Content Permission Form (http://journals.plos.org/plosone/s/file?id=7c09/content-permission-form.pdf) and the following text:

"I request permission for the open-access journal PLOS ONE to publish XXX under the Creative Commons Attribution License (CCAL) CC BY 4.0 (http://creativecommons.org/licenses/by/4.0/). Please be aware that this license allows unrestricted use and distribution, even commercially, by third parties. Please reply and provide explicit written permission to publish XXX under a CC BY license and complete the attached form."

Please upload the completed Content Permission Form or other proof of granted permissions as an "Other" file with your submission.

In the table caption of the copyrighted figure, please include the following text: "Reprinted from [ref] under a CC BY license, with permission from [name of publisher], original copyright [original copyright year]."

2. If you are unable to obtain permission from the original copyright holder to publish these figures under the CC BY 4.0 license or if the copyright holder's requirements are incompatible with the CC BY 4.0 license, please either i) remove the figure or ii) supply a replacement figure that complies with the CC BY 4.0 license. Please check copyright information on all replacement figures and update the figure caption with source information. If applicable, please specify in the figure caption text when a figure is similar but not identical to the original image and is therefore for illustrative purposes only."

We have permission from the copyright holder and have included a completed Content Permission Form as a separate file in the re-submission, and we have updated the note below Table 1 as suggested.

“12. Please include captions for your Supporting Information files at the end of your manuscript, and update any in-text citations to match accordingly. Please see our Supporting Information guidelines for more information: http://journals.plos.org/plosone/s/supporting-information.

We have included the captions of the Supporting Information files at the end of the manuscript.

If the reviewer comments include a recommendation to cite specific previously published works, please review and evaluate these publications to determine whether they are relevant and should be cited. There is no requirement to cite these works unless the editor has indicated otherwise.”

Done. Thank you.

Response to Reviewer 1's Comments

"The authors should considere this two papaers:
1. Family and Religion in Luba Life: Centrality, Pervasiveness, Change and Continuity Tshilemalema Mukenge (AUTHOR)
2. Pouvoir, territorialité et conflictualité au Grand Kasaï (République démocratique du Congo) Power, territoriality and conflicts in Large Kasaï (Democratic Republic of Congo) Kabata Kabamba (AUTHOR)
The two paper wil help to reinforce the similarities explanations between IDPs and host communities, and will shed some light on the IDPs preferences as well."

Thank you for your suggestion. We have read both papers.

The first reference (Mukenge 2010) examines the role of the family unit among the Luba tribe. While interesting, it does not align well with this study. We thus follow the Editor's suggestion and do not refer to it in the manuscript.

The second reference (Kabamba 2018) provides an overview and analysis of the persistent conflicts in the Kasai region, which is relevant for this paper. We now refer to it in "S1 Appendix. Conflict and Displacement in the Greater Kasai."

Response to Reviewer 2's Comments

"This study is an interesting report on factors driving the migratory preferences of Congolese. It contributes important insights to policymakers and practitioners working on migration. Overall, this work should be published in PlosOne. I think the MS could benefit from some reframing and added clarity in the text because there are meaningful differences among subpopulations (displaced versus non), as well as fixing of minor errors. I put some suggestions for additional analyses that should either be added to the main text or put into the appendix, depending on what they result in."

Thank you for the kind words and your valuable comments, which we have incorporated into the manuscript. We respond to each comment below.

"Framing/Contribution
I have some reservations about how the study is presented. It feels somewhat like the authors were struggling to cut words, and ended up cutting too much. Perhaps this also led them to frame the paper in what may have been the most parsimonious presentation, but to me, the current framing doesn't do justice to the study sample nor its findings.
I was confused for quite a while as to what types of people this study is about. The introductory framing leads the reader to believe that the paper will be about refugees and/or displaced persons seeking international integration. After reading the manuscript I thought it was about IDPs, but understood it also had some non-displaced persons mixed in. Yet, from the SI materials I found out that the sample actually comprises refugees, IDPs, and those who remained despite conflict. This really needs to be made clear in the main text of the MS and I think it needs to be more clearly addressed even in the abstract and introduction. How do these three groups differ from one another and how are they the same, especially with regards to the experimental outcomes?
I note this because the framing lumps IDPs with refugees and I question if this is a reasonable generalization to make. IDPs are a special group differentiated first, by the fact that they remain in their own country where they share a language (or usually at least some linguistic similarities) and cultural ties to a higher degree at least than they would had they crossed an international border (or at least this is the most likely case); and second, by the fact that they may be those who couldn't afford to go abroad - so they may be poorer and less educated than refugees. For instance, most refugees who came to Europe during the 2015 "crisis" are single males from wealthier backgrounds compared to those who remained in the region or were internally displaced. It seems also like those who fled abroad might have been more likely to actually experience some of these situations firsthand and especially things like that the community does not speak one's mother tongue.
The authors run an analysis of displaced versus not, what about returned refugees versus IDPs versus those who remained? I would like to see the results for actual refugees compared to not displaced as well to confirm all the claims that they are not different are confirmed.
We also already see in the SI file that displaced persons (lumping refugees and IDPs together)

are indeed diff from those who were never displaced. The latter are not significantly affected by being allowed at community meetings nor by relatives in the area. The MS says these differences are not significant but I couldn't find an actual test to verify that. Regardless if the two groups are not significantly different from each another, (this could be due to lack of power since the non-displaced sample is small), the MS should be more honest about what it's studying and what it finds. These subgroup differences should not simply be ignored.
This is important mainly because one could question how much fleeing from violence affects the outcomes at all and whether some of the preferences are just preferences about migration in general? The sample is largely suffering from a lack of regular income, so available jobs seems to be a desirable feature for all, regardless of displacement or not. The authors might want to reframe around these distinctions. For the sub sample of non-displaced, anything that is sig seems to matter for non displaced people as well as displaced. For displaced persons, other factors like family and attending village meetings are important when they are not for non-displaced persons. What might this indicate? Perhaps looking at the mechanisms by subgroups of IDPs, refugees, and those who remained would be helpful?"

We agree with the Reviewer. This study collected data from IDPs, returned IDPs, repatriated refugees and host community members (those without a displacement history). We had initially only separated out displaced and non-displaced respondents to keep the manuscript short. In response to your comments, and similar ones by Reviewer 3:

We have edited the text throughout and are now clear from the start of the manuscript that this study is about people displaced by the conflict - IDPs, returned IDPs, and repatriated refugees - and members of the host community.

We have created a summary table (Table S3-2), which provides sample characteristics across the four household types and an F-test whether any group is significantly different from the others.

We have updated the related results figure (Figure 4), which now splits up the AMCE effects by host community, IDPs, repatriated refugees, and returned IDPs. The full numeric results are provided in the appendix in Table S4-3.

Finally, we also formally test whether there are significant differences in the AMCEs across subgroups (see Table S4-5 in the appendix). We find that IDPs have stronger negative views about host communities in which they cannot work or not able to join the church compared to hosts. Repatriated refugees seem to value relatives in the community to a larger extent than host community members. There are no significant differences between returned IDPs and hosts, or between different populations with displacement history. We refer to this on page 8 of the manuscript.

"An additional robustness check for the experimental analysis would be to show results with controls for all potential differences, including one for IDPs, refugee, and never displaced but also the other indicators of household economics, gender, etc. Demonstrate how these potential differences don't matter for the experiment. Table S5 doesn't really accomplish this. But also since the authors have this information already separated out, the reporting of sample characteristics in the MS should be broken down by those who fled and those who did not, or even more preferably, IDPs, refugees, and never displaced."

Thank you. We have now added Table S3-2, which provides sample characteristics across the four household types. For each characteristic, we report an F-test assessing whether the means significantly differ across the household types; while there are statistical differences, the substantive differences are small.

In addition, we have undertaken the robustness test suggested by the Reviewer. Figures S6-4 and S6-5 replicate Figures 2 and 4, respectively, while controlling for a wide set of covariates (demographic, socio-economic, and health-related). The results are similar to the main findings and are now reported in the subsection 'Robustness' (page 8 of the manuscript).

"Experimental design:
The design could use some more explanation of how realistic these hypothetical safe havens to flee to are. How often do villages hold meetings and collect preferences of villagers? And how common is it that they would bar residents (or even that they would allow temporary residents to attend??) Do all respondents actually have family they could move to? Might not all their family live in the same village? Would a church really deny other Christians from participating? What kind of church is that? Along these lines: Are all respondents Christian? There's nothing on religion or how often people attend church. That seems bizarre given that church attendance is an experimental arm."

It is indeed important that the experiment's hypothetical profiles are realistic. During the design phase, we undertook much effort to be sure that this is the case. The attributes and levels are the result of many interactions with UNHCR and other field partners operating in the Kasai region. Two of the authors have over fifteen years of fieldwork experience in the DR Congo. Most importantly, the attributes and levels were carefully piloted during the five months prior to the survey. Between June and October 2022, we conducted two pilot studies, which included detailed debriefings with respondents to assess the importance, relevance and plausibility of the attributes, levels and drawings. Based on these pilots, attributes and levels were deleted, added, or revised. We now describe this process more carefully in the main text (page 5).

The study was indeed largely silent about the context around the experiment's attributes. In response, we now provide this information, when available in the household survey, in Table S3-1. For example, we now provide information about: village meetings (they happen regularly, 26% of respondents attended a community meeting the preceding year), work (50% of respondents was economically active in the 7 days prior to the survey), language diversity (32% do not speak Tshiluba, the main local language, as their mother tongue), and religion (90% of

respondents are Christian but there is much variation within Christianity (23% of respondents are Protestant, 15% Catholic, 25% Revivalist, etc.), and 62% goes to church at least once per week). Family networks in Kasai span many villages (marriage being an important reason) – but we do not have data from the survey to present. Nor do we have empirical evidence – beyond anecdotal evidence from field experience and interactions with UNHCR – that also recently arrived may participate in village meetings. On page 4 and 5, we now discuss the context around the experiment's attributes and refer to Table S3-1.

"The term "coethnic" is not well defined nor theorized. All three attributes of co-linguists, relatives, and co-religion can be considered to be coethnics. Why are there no indicators for language or religion in the samples? Particularly if language group is how ethnicity is defined in Congo - why are there no analyses controlling for ethnic group or language? Given that the experiment manipulates language, is it likely for some language groups that no one in a village would speak their language? Some African languages are very close to others, so this may not be a big problem. Minority language native speakers are also probably more likely to speak another more popular language. This begs the question: what is shared language doing in the experiment and how does this vary across sub populations?"

In this study's context, the Democratic Republic of Congo, ethnicity is indeed based on one's mother tongue. We thank the Reviewer for emphasizing the importance of clarity and precision on this point. What we have done in response is the following:

In the introduction, we note that in the DRC, ethnicity is based on an individual's mother tongue. Subsequently, we shift the focus from ethnicity to language ties and shared language, which corresponds with the attribute used in the conjoint experiment.

We now provide summary information about religion and language in Table S3-1, which we refer to and discuss on page 4.

Empirically, in the appendix, we replicate results controlling for a wide set of covariates – including respondent's religion and language – and obtain similar results (Figures S6-4 and S6-5). We also undertake a subgroup analysis, and do not find significant differences across Tshiluba speakers and other mother tongues (Figures S6-6). These results are now reported in the subsection 'Robustness' (page 8).

"The pictures accompanying the experiment don't just depict the points about lack of jobs or family or ability to get into meetings and churches, but also loneliness. They're a bit misleading from the main trigger/mechanism because they emphasize a person being left all alone. Like even if there aren't jobs, one could still volunteer help. Even if there's no family, a person might be able to make friends. One robustness check here would be to see if the illiterate (who maybe relied more on the pictures) differ from those who are literate. But, for instance, the arms on attending church and village meetings are also just about exclusion. For meetings - is it about not being welcome or about not being able to join in decisions? The authors attempt to address

some of this in the section on mechanisms, but the logic behind this section is not well explained. A clearer explanation of these analyses and the motivation for them is needed. Be clear how this section addresses weaknesses in the design."

We agree with the Reviewer that the drawings present more than only the one attribute level that we aim to highlight. We are confident, however, that respondents interpret the drawing as highlighting the intended attribute. During the experiment, enumerators placed the randomly selected attribute levels in front of the respondent and presented each profile by holding up the corresponding drawings while verbally stating the attribute level. In addition, we carefully piloted the drawings prior to the survey: each drawing was shown to respondents without additional text or sound and respondents were asked to interpret them.

We thank the Reviewer for suggesting a robustness check. In "S6 Appendix. Robustness Tests.", we now present results of a subgroup analysis by respondents' literacy status. We do not find meaningful differences between literate and non-literate participants. We refer to this analysis on page 8.

Finally, we have revised the language in the mechanisms section (page 6), clarifying the analyses and their motivation, which we believe has strengthened the presentation and argument of the section.

"But I also worry that since the three experimental manipulations of church, family, and shared language all get at social integration whereas there is only one arm for economic and political integration, so that claiming these other two mattered more could be problematic. Couldn't it be that each social integration arm is overlapping with the other two in its effects? So some of the effect is being soaked up by the other aspect(s)? Comparing which matters more - that your relatives are there or the community speaks your language could contradict somewhat. Your relatives may be expected to speak your language. So if the respondent gets the arm with few speak their language but their relatives are there, this detracts/weakens the effects of the shared language arm. Can the authors convincingly address this? Otherwise they may want to do away with the claim that economic and political integration matter more than social.

Perhaps this final point could be addressed by checking ACIEs between the experimental arms? Are any of the arms enhanced by the others? It seems shared language will matter community meetings and church, for example."

Church, family, and shared language indeed capture three social dimensions that may conceptually overlap. However, from our pilot in the Kasai region, we learned that: i) each dimension is important, and ii) they reflect distinct aspects of social integration. Empirically, these three attributes are independently randomized, so the estimated effects are averaged over all possible realizations of the other attributes.

We thank the Reviewer for the suggestion to examine potential interactions between treatment arms. These results are now presented in "S5 Appendix. Interactions between Treatment Arms". Overall, we find no evidence of interaction effects with one exception: the ability to work and the ability to attend church mutually reinforce each other. We refer to this analysis and its findings in the main text (page 7).

"Are figures 3 abd 4 diff? 4 should show us something on the displaced versus not but I think it's the same figure."

Thank you for catching this error, for which we apologize. We have now included the correct figures.

"The authors should also make the pre-analysis plan available. This manuscript is not anonymized so there's no reason it shouldn't be provided, but another approach could be an anonymized version within the SI materials."

At the time of submission, we believed the manuscript had to be made anonymous. We have now included a link to the study's publicly available pre-analysis plan on page 3.

"Smaller issues:
- Page 4 has at least one typo calling the experiment a "joint" experiment."

We have corrected this typo.

"It also could use a look in terms of writing flow. Some of the points made there seem out of place - like voting for right wing parties and asylum applications. The link between that setting and the Congo seems rather tenuous."

Thank you. This comment refers to the following sentence in the original manuscript: "Electoral outcomes in destination countries have been found to influence the share of refugees who apply for asylum, with a higher share of votes for right-wing populist parties correlating with a decrease in asylum applications.". We agree that this example is not relevant to the context of our study and have therefore removed it. In addition, we have revised the text in this section – and throughout the manuscript – to improve writing flow.

"- Page 9 I think 90% is missing from the sentence for the thin lines …"

This was indeed missing and we have now included this information (page 7).

"- Food insecurity measure seems to be off - it should be a number between 0 and 7 as I understand the question but the max is reported as 35 and the mean is 11?"

Thanks for the careful read. We understand the confusion. The text in the original variable description table (Table S5) read: "Sum of days in the past 7 days that household members had to rely on less preferred food, borrow food, reduce meals, limit portion sizes or restrict adult food consumption" (and then indeed had a minimum 0 and maximum 35).

In fact, we make use of the World Food Program's Reduced Coping Strategies Index, which measures household food insecurity over the past seven days. Respondents report how many days the household had to: 1) rely on less preferred food, 2) borrow food, 3) reduce meals, 4) limit portion sizes, 5) restrict adult food consumption. Each item is scored from 0 to 7, and the scores are summed to produce a total index ranging from 0 to 35.

We have now improved the description of this variable and several others in Table S3-1. In addition, we have made all data and instruments available online, including the survey, and added a reference to it on page 4. Finally, to help the reader, we have now also included a column in Table S3-1 indicating the question number in the survey.

- Sampling - what does most affected by displacement mean? Places where people fled or fled to or both?

We selected 27 of the 63 health zones in the Kasai region in collaboration with the UNHCR. Relying on UNCHR data, we selected health zones where the number of IDPs and returned IDPs were particularly high. We have edited the text in the manuscript to clarify this (page 4). We have also expanded the discussion about the selection of health zones in the Supplementary Information "S2 Appendix. Sampling Frame and Sample".

Response to Reviewer 3's Comments

"The main issue of the article is the forced correlation between refugee and IDPs. This leads to confusion and miss-led comparison about refugee studies and reasons to move, and the myriads of reasons sustaining their decision making (to flee, and to where) are not like those of displaced people. To review based the Kampala Convention and 951 Refugee Convention and its 1967 Protocol 951 Refugee Convention and its 1967 Protocol. With this, minor suggestions and changes are needed."

Thank you for the comments you added in the manuscript. We have copied & pasted them below and respond to each one by one, and by doing so also respond to the paragraph above:

Page 2: *"These are rather from leading international or humanitrain organisations, not directly from policymakers per si"*

Thank you. We have edited this in the text (now page 2).

Page 3: *"This would require more arguments, and discussion. Ethnic ties are fixed in which sense?"*

This relates to the sentence (page 3 in the original manuscript): "Although certain community characteristics, such as family or ethnic ties, are fixed, others can be actively leveraged to support the integration of displaced populations."

Indeed, our original wording was brief. In the social sciences, there is an extensive debate on this issue. On the one hand, primordialism largely views ethnic identity as innate, fixed and permanent. Constructivist and instrumentalist views, on the other hand, argue that ethnic identities can change; e.g., political developments can shape which identities get activated. It is beyond the scope of this paper to interact with this debate in-depth. In response to the Reviewer's comment, we have revised the text to indicate that in the Congolese context, several characteristics – such as language, which serves as a marker for ethnicity in the DRC – are "difficult to change" (now page 2).

We also note that, in response to a comment by Reviewer 2, we have been more careful throughout the manuscript regarding the term ethnicity, and now consistently refer to language ties – which corresponds with the attribute used in the conjoint experiment.

Page 4: *"Here is the questioning point: is this study about refugees or displaced people (IDPs). The suggestion here is to revisit the Kampla Convnetion and 951 Refugee Convention and its 1967 Protocol 951 Refugee Convention and its 1967 Protocol"*

This confusion was also raised by Reviewer 2. We do apologize. This study collected data from IDPs, returned IDPs, repatriated refugees and host community members (those without a displacement history). To keep the manuscript short, we had initially only separated these out

between displaced and non-displaced populations. In the revised manuscript, we now i) introduce all four groups, ii) edited the text throughout the manuscript from two to four displacement groups, and iii) updated the related results figure (Figure 4) as well as the tables with summary statistics and results (Tables S3-2, S4-5 and S6-2).

Page 4: *"The correlation here is forced. Refugee studies and reasons to move, and the myrad of reaons sustaining their decision making (to flee, and to where) are not similar to those of displaced poeple. To review based on the comment above"*

We agree with the Reviewer and have therefore separated out the displaced populations into three groups: i) those displaced within the countries border but have not yet returned (IDPs), ii) those that have (returned IDPs) and iii) those that crossed the border into Angola and have returned (repatriated refugees). Along with individuals that do not have a history of displacement – members of the host community – these four groups represent the complete set of displacement histories within the communities. By design, our survey collected data from all four groups. In contrast to the original version of the manuscript, we now discuss groups explicitly, and present results for each separately. For more details, we refer the Reviewer to our response above regarding a similar comment from Reviewer 2.

Response to Reviewer 4's Comments

"What characteristics do displaced people value in looking for a place to settle? The authors use a conjoint survey experiment to ask about 2000 respondents living in a region of the Democratic Republic of the Congo that has been impacted by internal forced displacement. 74% of these respondents are internally displaced people (IDPs). In the conjoint, they examine the following 5 attributes: whether work is available or not, a local church or not, family members or not, local language or not, and ability to participate in village meetings or not. This is an impressive data collection effort, and I am curious about the other insights that this survey might provide."

Thank you. Indeed, the survey represents a considerable effort in an under-researched area. To maximize the use of the study's data, we have made the anonymized dataset publicly available at Harvard Dataverse (https://doi.org/10.7910/DVN/PHKNSU).

Below, we respond individually to your useful comments and suggestions.

"With respect to the main conjoint experiment, I suggest that the authors need to be more precise about the interpretation of their findings and do formal hypothesis testing to verify their claims. Although the authors claim that they find work opportunities is most important, more than coethnicity or family, the actual results in Figure 2 show that all 5 attributes may be equally important — the AMCE for work seems slightly more important than the others but that is not formally tested.

The conjoint has a very simple setup. Within each of the 5 attributes, there are only two levels and one level is always obviously preferred to the other — i.e. opportunity to work is better than no work, local church is better than no church, having relatives is better than not, having a common language is better than not, being able to attend village meetings is better than not — holding all the other attributes constant. The marginal means shown in Fig. S3 and the conjoint results with a continuous preference measure in Fig. S4 seem to the show the same as the main result.

Thus, the important comparisons are not within each attribute, but rather across attributes — comparing the importance of work against church against relatives, etc. But from Figure 2, the CIs seem to overlap, perhaps work stands out a little, but that will need to be formally shown. Are the differences between the estimates statistically significant?

It may be useful for the authors to reference this 2022 AJPS article: "What Do We Learn about Voter Preferences from Conjoint Experiments?" by Abramson, Kocak, and Magazinnik to how to interpret the AMCE precisely."

The Reviewer is correct that the key comparisons involve not only within-attribute but also across attribute differences. In response, we have added a formal test examining whether the AMCEs for each attributes differ significantly from the other attributes (Table S4-4 in the appendix).

Consistent with our initial visual inspection in Figure 2, work opportunities emerge as the most important attribute, with differences that are statistically significant when compared to each of the other attributes. While there are differences in the magnitude of the AMCEs for the other attributes, we cannot conclude that any one of them is more important than the others. We now discuss this in the results section on page 7.

The suggested reading (Abramson et al 2022) is indeed of methodological importance given the difficulties to aggregate population preferences based on conjoint experiments. We refer to the paper when discussing the usage of marginal means and AMCEs on page PPPP.

"Originally, when I started reading this paper, I had generalizability concerns — can the authors discuss how these findings generalize to other displacement contexts and from IDPs to refugees, for example? But seeing these results, I wonder if they would extend to many (non-displaced) populations, not just a vulnerable one experiencing insecurity. Indeed, the authors find similar results comparing the IDPs with the host community members in their sample."

Thank you. We agree that generalizability is an important consideration, and we believe the Reviewer's intuition may be correct. Two aspects of our findings suggest broader relevance. First, preferences for community attributes are very similar across respondent types. Second, the underlying mechanisms – economic security, agency, and belonging – are not specific to displaced populations. This suggests our findings may be broadly relevant to populations facing hardship. We now make this point in the conclusion on page 9, and on the same page, also suggest that similar studies should be conducted in other contexts.

"I also wonder why the authors chose to focus on these 5 attributes. There are others that this literature discusses notably, availability of land, access to markets, and access to humanitarian aid, for example. Of course any conjoint, especially if the authors needed to keep it simple, need to prioritize some attributes over others, but I wonder if adding either of these other important attributes would shift the results so that some attributes would stand out — for example a stronger preference for work rather than aid."

The attributes used in this conjoint experiment, indeed, do not represent the full range of host-community characteristics of interest. Given the context, we limited the design to five attributes as prior experience with similar experiments in the DRC – where most of the population has low education levels – indicated that five attributes is optimal. The selection process unfolded as follows: an initial set of attributes and levels was developed in consultation with UNHCR and other field partners active in the Kasai region. We then conducted two pilot studies in the field, involving: i) detailed discussions with villagers to assess the relevance and applicability of each attribute and level, and ii) checks on how the attributes, levels and drawings were interpreted. Based on these pilots, attributes and levels were deleted, added, or revised, and the accompanying drawings were likewise adjusted. We now describe this process more carefully in the main text (page 5).

The current five attributes and related levels were those that our partners and respondents indicated were most important and relevant in the Kasai context. Other attributes, however, are likely also relevant in this and other contexts. We now explicitly state this in the conclusion (page 9).

"Finally, I think the authors can expand Section 2: Preferences for Host Communities more so that it does more than simply list and summarize existing studies. This section can highlight the unique contributions of this paper as well as list the hypotheses."

Thank you. We have now revised Section 2 and explicitly list the study's contribution and pre-registered hypotheses, including a link to our pre-analysis plan (page 3).

Our pre-analysis plan also indicated that we would investigate results by the displacement history of households. We mention this now explicitly on page 3, and do so in the Section "Preferences by Displacement Experience".

END
